# Inhibition of ATM kinase upregulates levels of cell death induced by cannabidiol and γ-irradiation in human glioblastoma cells

**DOI:** 10.18632/oncotarget.26582

**Published:** 2019-01-25

**Authors:** Vladimir N. Ivanov, Jinhua Wu, Tony J.C. Wang, Tom K. Hei

**Affiliations:** ^1^ Center for Radiological Research, Department of Radiation Oncology, Vagelos College of Physicians and Surgeons, Columbia University, New York, NY 10032, USA

**Keywords:** glioblastoma, cannabidiol, radiotherapy, ATM kinase, TRAIL-R2

## Abstract

Despite advances in glioblastoma (GBM) therapy, prognosis of the disease remains poor with a low survival rate. Cannabidiol (CBD) can induce cell death and enhance radiosensitivity of GBM but not normal astrocytes. Inhibition of ATM kinase is an alternative mechanism for radiosensitization of cancer cells. In this study, we increased the cytotoxic effects of the combination of CBD and γ-irradiation in GBM cells through additional inhibition of ATM kinase with KU60019, a small molecule inhibitor of ATM kinase. We observed in GBM cells treated by CBD, γ-irradiation and KU60019 high levels of apoptosis together with strong upregulation of the percentage of G2/M-arrested cells, blockade of cell proliferation and a massive production of pro-inflammatory cytokines. Overall, these changes caused both apoptotic and non-apoptotic inflammation-linked cell death. Furthermore, via JNK-AP1 activation in concert with active NF-κB, CBD upregulated gene and protein expression of DR5/TRAIL-R2 and sensitize GBM cells to TRAIL-induced apoptosis. In contrast, CBD notably decreased in GBM surface levels of PD-L1, a critical immune checkpoint agent for T-lymphocytes. We also used in the present study TS543 human proneural glioma cells that were grown as spheroid culture. TS543 neurospheres exhibited dramatic sensitivity to CBD-mediated killing that was additionally increased in combination with γ-irradiation and KU60019. In conclusion, treatment of human GBM by the triple combination (CBD, γ-irradiation and KU60019) could significantly increase cell death levels *in vitro* and potentially improve the therapeutic ratio of GBM.

## INTRODUCTION

Human gliomas are the most prevalent primary tumors of the central nervous system (16% of all primary brain tumors). Approximately 54% of all newly diagnosed gliomas are glioblastoma (GBM), the most malignant and lethal primary brain cancer in adults. Despite advances in GBM therapy, outcomes remain poor with a median survival rate of 12-15 months after initial diagnosis [1, 2]. The standard of care for GBMs includes maximal safe surgical resection followed by external beam radiotherapy (with a total dose of 60 Gy) and concurrent temozolomide (as a supplemental DNA-damage agent), followed by 6 to 12 months of adjuvant treatment with temozolomide [3]. However, the vast majority of GBMs eventually relapse and new treatment modalities are desperately needed. Normal glial cells exhibit a substantial degree of radioresistance, while adult neurons, endothelial cells, oligodendrocyte precursor cells (OPC) and neural stem/progenitor cells (NSC/NPC), are highly sensitive to ionizing radiation alone or in combination with chemotherapy. Clinical observations and animal experiments demonstrate that cranial irradiation causes substantial cognitive deficits due to neuronal and endothelial cell damage, as well as inhibition of the proliferation and death of OPC and NSC/NPC [4–11]. Thus, advances in GBM treatment must not only be efficacious but significantly safer.

At the beginning of this century, killing GBM by cannabinoids *in vitro* and in animal experiments was elucidated in numerous studies [12–15]. Additional investigations also confirmed the cytotoxic role of cannabinoids for several other types of cancer [16–18]. A number of studies with GBM cells demonstrated the efficiency of combined treatments of cannabinoids together with γ-irradiation both in cell culture and in animal experiments [19–21]. The advantage of substituting a single modality treatment with a combination of treatments is the possibility to minimize toxicity and to optimize doses of ionizing radiation. On the other hand, drugs in combination with radiotherapy are often used at a lower dose than in monotherapy. Combined therapy may allow attacking several signaling pathways in GBMs and potentially overcomes a characteristic feature of GBMs to develop treatment resistance. Several former studies demonstrated a leading role for ATM kinase in regulation of radioresistance of cancer cells [22–26]. Specific pharmacological inhibitors of ATM kinase activity are currently under preclinical and clinical investigation for cancer treatment, including upregulation of radiosensitivity of tumors [25]. Based on previous studies of the regulation of cell death signaling in GBM after combined treatment with cannabidiol (CBD) and γ-irradiation [19, 21], we evaluated in the present study the impact of a small molecule inhibitor of ATM kinase KU60019 [26] as the third component of combined treatment to increase the efficacy of GBM killing.

## RESULTS

### Signaling pathways induced by combined treatments with CBD, the ATM kinase inhibitor KU600199 and γ-irradiation in U87MG human GBM cells

**Figure 1:**
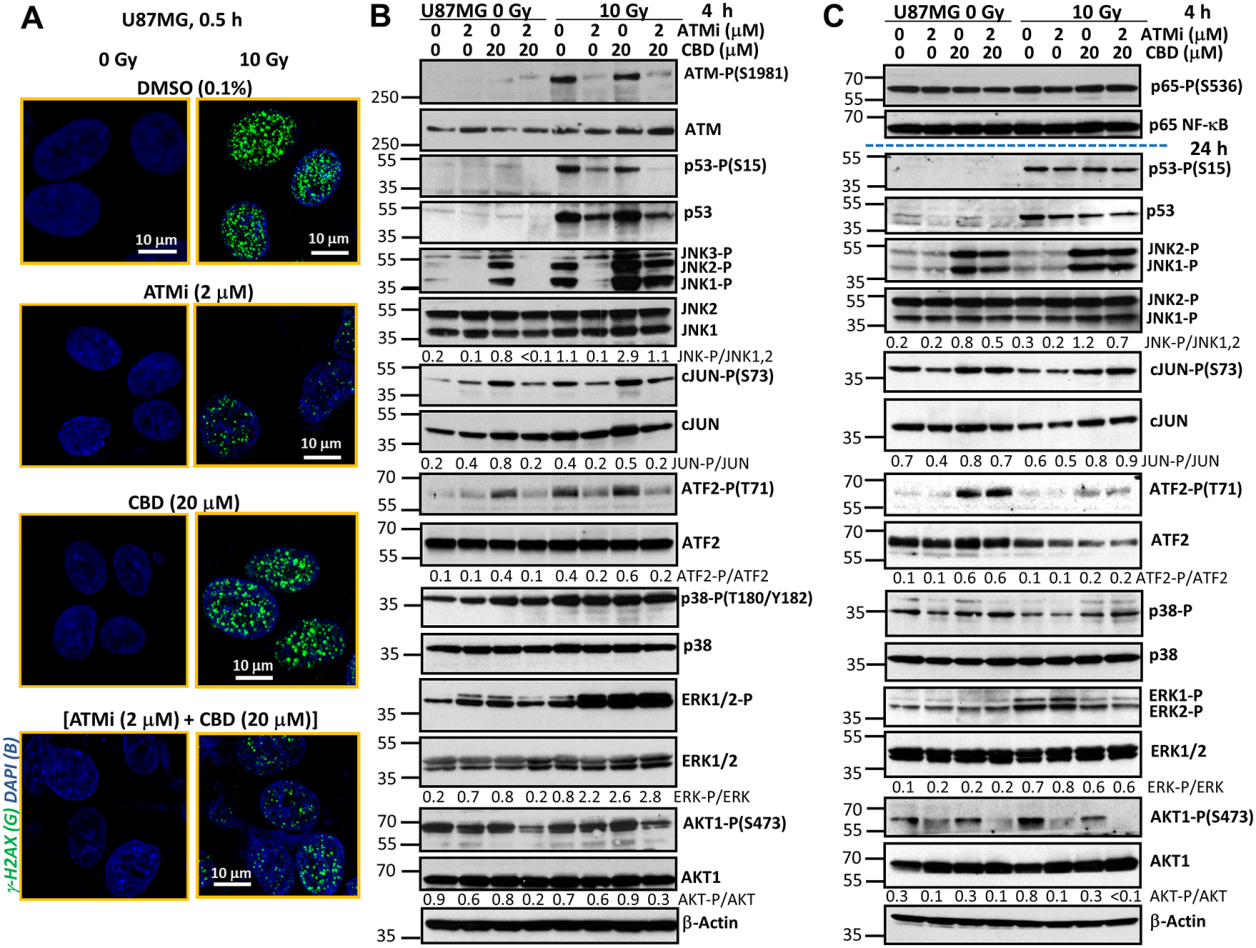
Effects of ATM kinase inhibition on radiation response of U87MG GBM cells **(A)** Effects of γ-irradiation (10 Gy), alone or together with cannabidiol (CBD, 10 μM in 0.1% DMSO), the ATM kinase inhibitor (ATMi) KU60019 (2 μM) in 0.1% DMSO on induction of DNA DSB in the nuclei of U87MG cells 0.5 h after treatment. DSB foci formation was determined using immunostaining with anti-H2AX-P-(S139) Ab (green) and DAPI staining of DNA (blue) that was followed by confocal microscopy. Bar = 10 μm. **(B and C)** Changes in the patterns of signaling proteins after treatment of U87MG cells with ATMi (2 μM) and CBD (20 μM), alone or in combination, followed by γ-irradiation at 10 Gy. CBD, ATMi and 0.1% DMSO (vehicle) were added to the cell cultures 30 min before irradiation. Western blot analysis of indicated signaling proteins from U87MG cells was performed 4 h and 24 h after treatments.

ATM kinase plays a crucial role as a sensor of double-strand breaks in genomic DNA and as the initiator of DNA repair after nuclear ionizing irradiation. Furthermore, active ATM kinase affects numerous cytoplasmic targets that regulate cell signaling pathways and general cell survival [24]. Since ATM kinase activation upon γ-irradiation regulates a balance between cell survival and death pathways, we used the ATM kinase inhibitor KU60019 [26] to investigate its effects in combination with CBD on radiosensitization of cancer cells. As expected, our initial experiments demonstrated efficient phosphorylation of histone H2AX after γ-irradiation of U87MG GBM cells, while CBD (20 μM) pretreatment did not notably affect basal levels, as well as radiation-induced ATM-mediated γ-H2AX foci formation (Figure 1A). On the other hand, we observed substantial suppression of γ-H2AX foci formation after γ-irradiation in the presence of the ATM kinase inhibitor (ATMi) KU60019 (1-2 μM). Finally, the triple combination of CBD, ATMi, and γ-irradiation demonstrated a strong downregulation of foci formation (Figure 1A), allowing to maintain the DNA damage conditions. The efficiency of DNA repair 6 h after the initial treatment was reflected by a strong decrease of γ-H2AX foci formation in the nuclei of the control irradiated cells and small changes in ATMi- or (ATMi+CBD)-treated irradiated cells (data not shown).

Western blot analysis confirmed the specificity of the ATMi KU60019 (at concentration 1-2 μM) for suppression of γ-irradiation-induced ATM kinase activation in U87MG GBM cells 4 h after treatment (Figure 1B). As a result of this inhibition, a characteristic consequence of DNA-damage-induced ATM kinase activation, ATM-dependent Ser-15 phosphorylation, and stabilization of p53 protein levels [22] was also suppressed in GBM cells (Figure 1B). CBD treatment, alone or in combination with γ-irradiation strongly affects the activation of MAPKs, including JNK1/2, MAPK p38, and ERK1/2, in GBM cells [21]. We addressed a probable interference of ATM-mediated signaling and CBD-induced activation of MAP kinases. We confirmed previously described data that JNK1/2 phosphorylation/activation was very strong 4 h after treatment with CBD and γ-irradiation, alone or, especially in combination, in U87MG cells (Figure 1B). Partial but significant suppression of JNK1/2 phosphorylation in the presence of KU60019 (1-2 μM) further decreased activation of JNK downstream targets, cJUN-P(S73) and ATF2-P(T71), in CBD-treated non-irradiated or irradiated GBM cells 4 h after treatment (Figure 1B). ATMi did not affect MAPK p38 phosphorylation at this time point. It should be mentioned that ATF2-T71 phosphorylation is directed mostly by JNK, rather than MAPK p38 in U87MG cells [21], correlating with JNK activity. On the other hand, a direct ATM-driven phosphorylation of ATF2-Ser490/498 was elucidated previously that was important in the regulation of DNA damage response [27]. The negative effects of ATMi on phosphorylation of p53, JNK, cJUN and ATF2 were almost not pronounced 24 h after treatment (Figure 1C).

ERK1/2 phosphorylation was notably increased 4 h after γ-irradiation in the presence of ATMi, CBD or both and then gradually decreased 24 h after treatment (Figure 1B and 1C). In contrast, phosphorylation-activation of AKT1-Ser473 was partially suppressed in the presence of ATMi, in both non-irradiated and irradiated U87MG cells 4 h and, especially, 24 h after treatment. Levels of phosphorylated NF-κB p65-P(S536) and total NF-κB p65 were relatively stable 4 h - 24 h after treatment (Figure 1C).

Taken together, these results demonstrate that CBD treatment induces pronounced changes in activities of several crucial protein kinases and their corresponding targets in the non-irradiated and irradiated cells that regulate cell proliferation, survival, and death. Additional modulation of these activities by cotreatment with ATMi in concert with simultaneous suppression of DNA repair could dramatically change cell fate. Although suppression of ATM kinase activation downregulated p53-mediated cell death pathways such as what in U87MG cells, it also opened alternative possibilities for regulation of cell death and survival [28, 29].

### Inhibition of ATM kinase increased the death of GBM cells induced by combined treatment of γ-irradiation and CBD

**Figure 2:**
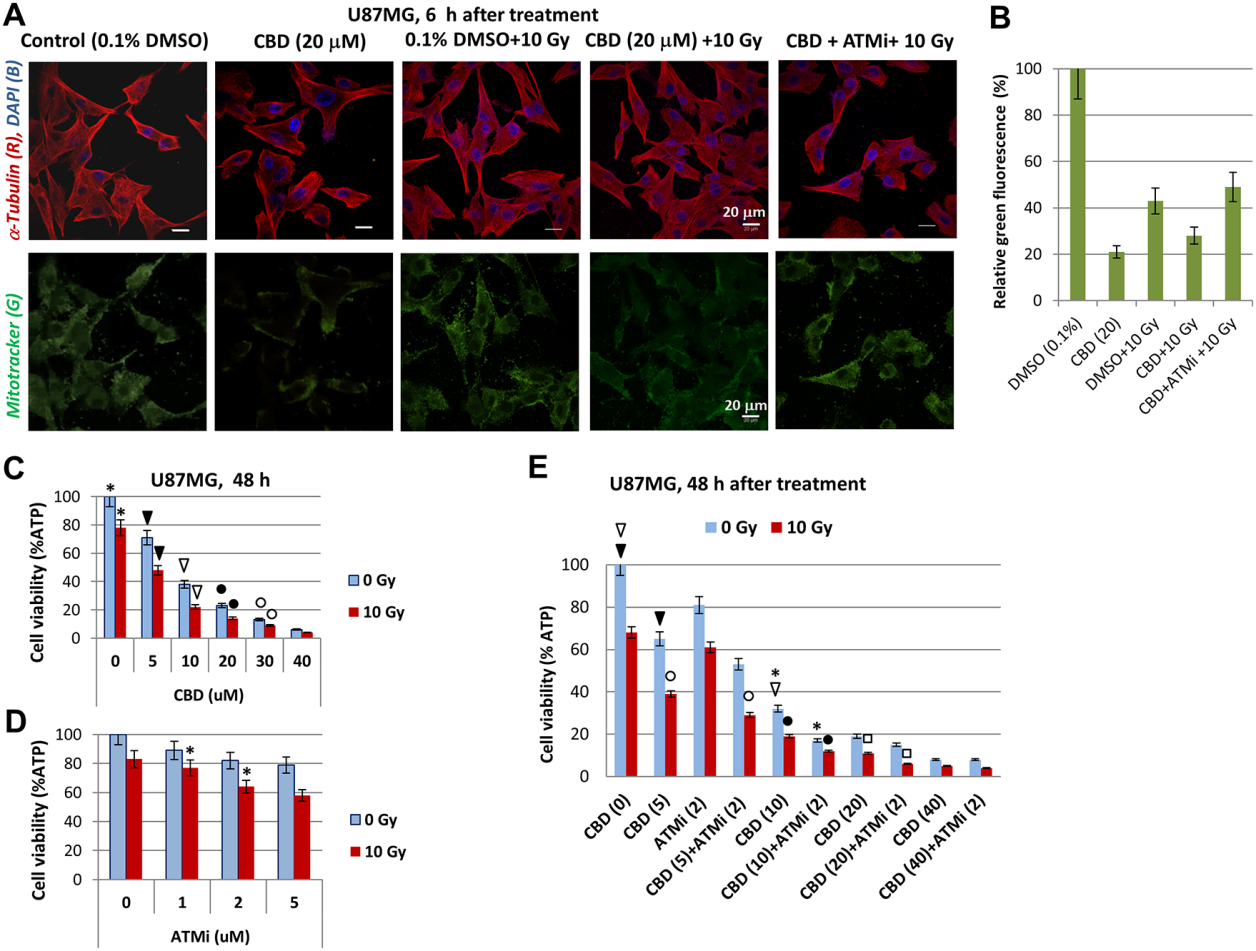
CBD, alone or in combination with ATMi and γ-irradiation (10 Gy) decreased the viability of U87MG cells **(A)** Effects of CBD (10 μM), ATMi (2 μM) and γ-irradiation (10 Gy) on functional activity of mitochondria in U87MG cells detected 6 h after treatment using Mitotracker (green) incubation and confocal microscopy. Additionally, cells were stained with anti-α-Tubulin mAb (red) and DNA-specific DAPI (blue). Bar = 20 μm. **(B)** Relative levels of Mitotracker-induced green fluorescence after indicated treatments of U87MG cells. Pooled results of three independent experiments 48 h after treatments are shown. Error bars represent means ±S.D. (*p* < 0.05, Student's t-test). **(C-E)** Effects of CBD (5-40 μM) and ATMi (1-5 μM), alone or in combination with γ-irradiation (10 Gy) on U87MG cell viability using the determination of intracellular ATP via ATP-dependent luminescence intensity 48 h after treatment. Combinations of CBD (5-20 μM), ATMi (2 μM) and γ-irradiation (10 Gy) were used in the panel E. Stars, arrows, circles, and squares indicate significant differences in the cell viability after specified treatments.

Following up on the previous finding that CBD induced production of ROS and damaged mitochondrial function in human glioblastomas [14, 30, 31], we performed functional staining of active mitochondria with MitoTracker (green) to ascertain if combined treatment in the presence of ATMi affected normal mitochondrial function in U87MG cells. As shown in Figure 2A and 2B, normal mitochondrial function in U87MG cells was impaired 6 h after treatment with CBD (20 μM) alone or in combination with γ-irradiation (at 10 Gy). On the other hand, a combination of CBD, ATMi and γ-irradiation (10 Gy) negatively affected levels of mitochondrial activity 6 h after treatment, similarly with just a combination of CBD and 10 Gy, indicating that the early effects of ATMi on mitochondria were not sufficient to significantly change the action of CBD and γ-irradiation.

A massive detachment of dying GBM cells and a lower ratio of adherent live cells with changed shape was observed later at 48-72 h after treatment with CBD, alone or in combination with irradiation [21]. Precise quantitative determination of cell viability (using detection of ATP-dependent luciferase activity in CellTiter-Glo cell viability assay) demonstrated dose-dependent negative effects of CBD (5-40 μM) on glioblastoma cell survival that was additionally increased by irradiation at 10 Gy. For CBD doses of 5-40 μM, well-pronounced additive effects in combination with γ-irradiation were established, although the highest dose of CBD used (40 μM) by itself dramatically decreased cell viability with relatively small additional effects of γ-irradiation (Figure 2C).

We used the ATMi KU60019 to further investigate its general effects on radiosensitization of cancer cells [26], alone or in combination with CBD. Treatment with KU60019 (1-5 μM) resulted in a modest and dose-dependent decrease of U87MG cell viability 48 h after treatment (Figure 2D). Concurrent treatment with γ-radiation (10 Gy) increased the toxicity in an additive manner (Figure 2D). Doses of 1-2 μM KU60019 were selected for all subsequent experiments. Cell viability assays demonstrated that the presence of KU60019 (2 μM) significantly decreased the viability of U87MG cells 48 h after combined treatment with CBD (5-20 μM) and γ-irradiation (Figure 2E). However, there was the absence of significant negative effect of KU60019 on U87MG viability after combined treatment with the highest dose of CBD (40 μM) and γ-irradiation (10 Gy) (Figure 2E). Hence, prolonged effects of ATM kinase inhibition resulted in an increase of cell killing by CBD (at low and intermediate doses) and γ-irradiation.

**Figure 3:**
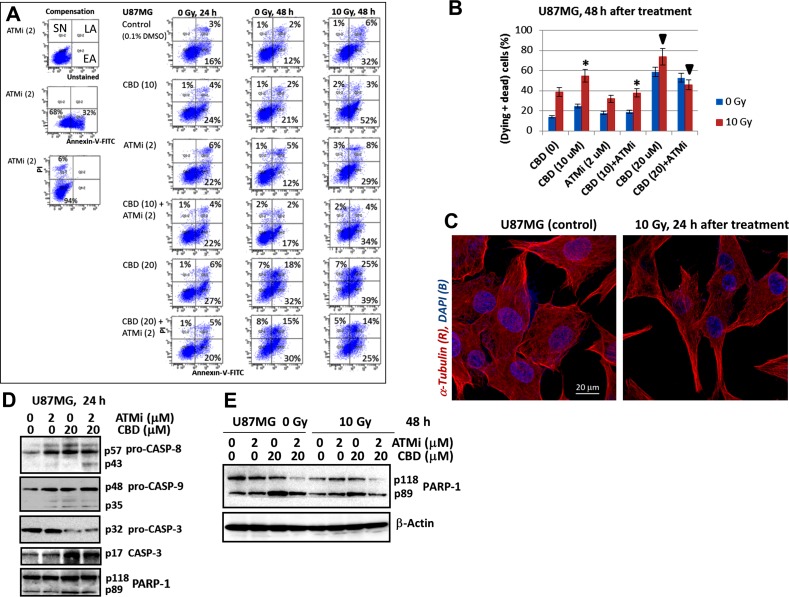
The apoptotic commitment of U87MG after treatment with CBD (10-20 μM), ATMi (2 μM) and γ-irradiation (10 Gy), alone or in combinations **(A and B)** Annexin-V-FITC and PI staining for determination of early apoptotic (EA), late apoptotic (LA) and secondary-necrotic (SN) GBM cells after indicated treatment was followed by the flow cytometry. Typical experiment **(A)** and pooled results of four independent experiments **(B)** using U87MG cells 24-48 h after indicated treatments are shown. Percentage of (dying + dead cells) included early apoptotic (EA), late apoptotic (LA) and secondary necrotic cells (SN). Error bars represent means ± S.D. (*p* < 0.05, Student's t-test). The stars and the arrows indicate significant differences between indicated cells after specified treatment. **(C)** The images of control and irradiated U87MG after immunostaining with α-Tubulin and DAPI followed by confocal microscopy are shown. **(D and E)** Western blot analysis of apoptotic marker proteins 24 h and 48 h after indicated treatments of U87MG cells.

We used (Annexin-V-FITC + PI) staining of U87MG cells and the flow cytometry to further distinguish between early apoptosis (EA), late apoptosis (LA) and secondary necrosis (SN) 24 h and 48 h after indicated treatment modalities (Figure 3A and 3B). CBD (10-20 μM) and γ-irradiation at 10 Gy effectively induced early and advanced phases of apoptosis in U87MG cells, while the presence of ATMi (KU60019) decreased CBD-induced apoptotic levels 48 h after treatment (Figure 3A). Figure 3B summarized the percentage of early apoptotic (dying) and late apoptotic/secondary necrotic (dead) cells 48 h after treatment. At this time point, the presence of ATMi significantly decreased apoptotic death levels induced by (CBD+10Gy) treatment, while total cell death levels were actually increased (see Figure 2E). It indicated that a non-apoptotic mechanism was probably involved in (CBD+ATMi+10Gy)-induced total cell death that dominated a decrease in apoptotic signaling. Only relative mild morphological changes were observed on confocal images of control and irradiated U87MG cells 24 h after treatment (Figure 3C).

An additional proof of apoptotic commitment, caspase-8 and caspase-9 activation (detected via the appearance of cleaved subunits) was at relatively low levels 24 h after treatment of U87MG cells, while caspase-3 activation (detected via the release of p17 subunit, as well as via caspase-3-mediated PARP-1 cleavage was well-pronounced (Figure 3D). Advanced apoptosis was observed 48 h after treatment. It was accompanied by an enhanced caspase-3-dependent cleavage of PARP-1 (with releasing the p89 cleaved subunit) after CBD treatment alone or with γ-irradiation. These data also demonstrated a decrease of levels of PARP-1 p89 cleaved subunit in the presence of ATMi (Figure 3E).

### Effects of combined treatment on cell cycle, proliferation and clonogenic survival of U87MG cells

**Figure 4:**
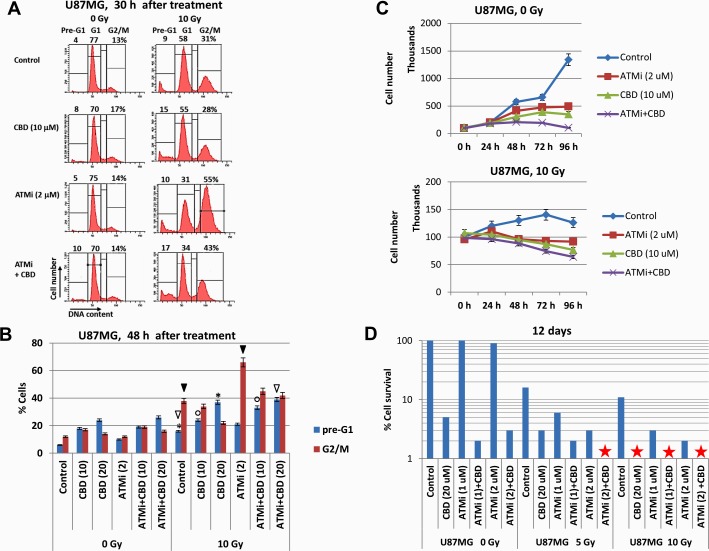
Analysis of cell cycle, cell growth, and cell death following treatment of U87MG GBM with the ATMi inhibitor KU60019, CBD and γ-irradiation, alone or in combination **(A and B)** Changes in the cell cycle and levels of death were determined following treatments of U87MG cells with CBD (10-20 μM), ATMi (2 μM) and γ-irradiation at 10 Gy, alone or in combination. Cell cycle-apoptosis analysis was performed 30 h and 48 h after indicated treatments. CBD, ATMi or their combination, as well as control 0.1% DMSO, were added 30 min before irradiation. The nuclei of control and treated cells were stained with PI and DNA content in cells was determined using the flow cytometry. The nuclei of apoptotic and secondary necrotic cells were localized in the pre-G1 region. A typical result 30 h after treatment is shown in the panel **(A)**. Pooled results of four independent experiments for U87MG 48 h after treatments are shown in panel **(B)**. Error bars represent means ±S.D. (*p* < 0.05, Student's t-test). The stars, arrows, and circles indicate significant differences in the pre-G1 and the G2/M levels between cells after specified treatment. **(C)** Kinetics of U87MG cell growth was monitored 0-96 h after indicated treatments: 0.1% DMSO (as control), ATMi (2 μM), CBD (10 μM), γ-irradiation (at 5 Gy), alone or in combination. **(D)** Clonogenic survival assay was performed for cell cultures after indicated treatments with 0.1% DMSO (as control), ATMi (1-2 μM), CBD (20 μM) or a combination of ATMi+CBD without or with γ-irradiation at 5 Gy and 10 Gy. The final number of cell clones was determined after 12 days of cell culture growth. A ratio of a number of clones of treated cells to a number of clones of control cells reflects cell survival (percentage). Stars indicate the absence of survived clones.

Next, we determined the effects of ATM inhibition, alone or in combination with γ-irradiation and CBD, on the regulation of the cell cycle, cell proliferation and clonogenic survival of U87MG cells. FACS analysis demonstrated, as expected, a strong increase in the percentage of G2/M-arrested cells 30 h after γ-irradiation, especially in the presence of ATMi (Figure 4A). Furthermore, two combined treatment modalities (CBD+10Gy irradiation) or (CBD+ATMi+10Gy), similarly and modestly increased the percentage of pre-G1 cells (that contained apoptotic and secondary necrotic cells) at 30 h (Figure 4A) and 48 h after treatment (Figure 4B). The percentage of GBM cells arrested at the G2/M phase was maximal at 48 h after treatment with (ATMi+10Gy irradiation), while after (ATMi+CBD+10Gy) treatment this ratio actually decreased with redistribution of cells between the pre-G1 and the G1 cell subpopulations (Figure 4A and 4B). In general, a high ratio of irreversibly arrested G2/M cells after suppressing the DNA repair predicted a prolonged cell death via a necrotic mechanism, such as necrotic mitotic catastrophe.

Cell growth kinetics clearly demonstrated diverse suppressive effects of CBD, ATMi, γ-irradiation and their combinations on the growth of U87MG cell culture (Figure 4C). Combined treatment of U87MG cells with ATMi+CBD+10Gy induced rapid growth retardation, due to suppression of cell proliferation and increased cell death (Figure 4C, the bottom panel). Finally, clonogenic survival assay of U87MG cells revealed 70% elimination of cell clones by combined treatment of ATMi (2 μM) + CBD (20 μM) in non-irradiated cells and the complete elimination of U87MG clones after additional γ-irradiation (at 5 Gy) (Figure 4D). Interestingly, a combination of [ATMi (2 μM) + CBD (20 μM) + γ-irradiation (5 Gy)] resulted in an equivalent cell toxicity to [CBD (20 μM) + γ-irradiation (10 Gy)], further indicating a radio-sensitizing role for ATMi in U87MG treatment (Figure 4D). These results confirmed the important role of ATM inhibition in the complete elimination of treated GBM cells. Our next task was to evaluate the levels of apoptotic and non-apoptotic cell death after combined treatment in two additional GBM cell lines.

### Survival response of T98G and U118 GBM cells to combined treatment

**Figure 5:**
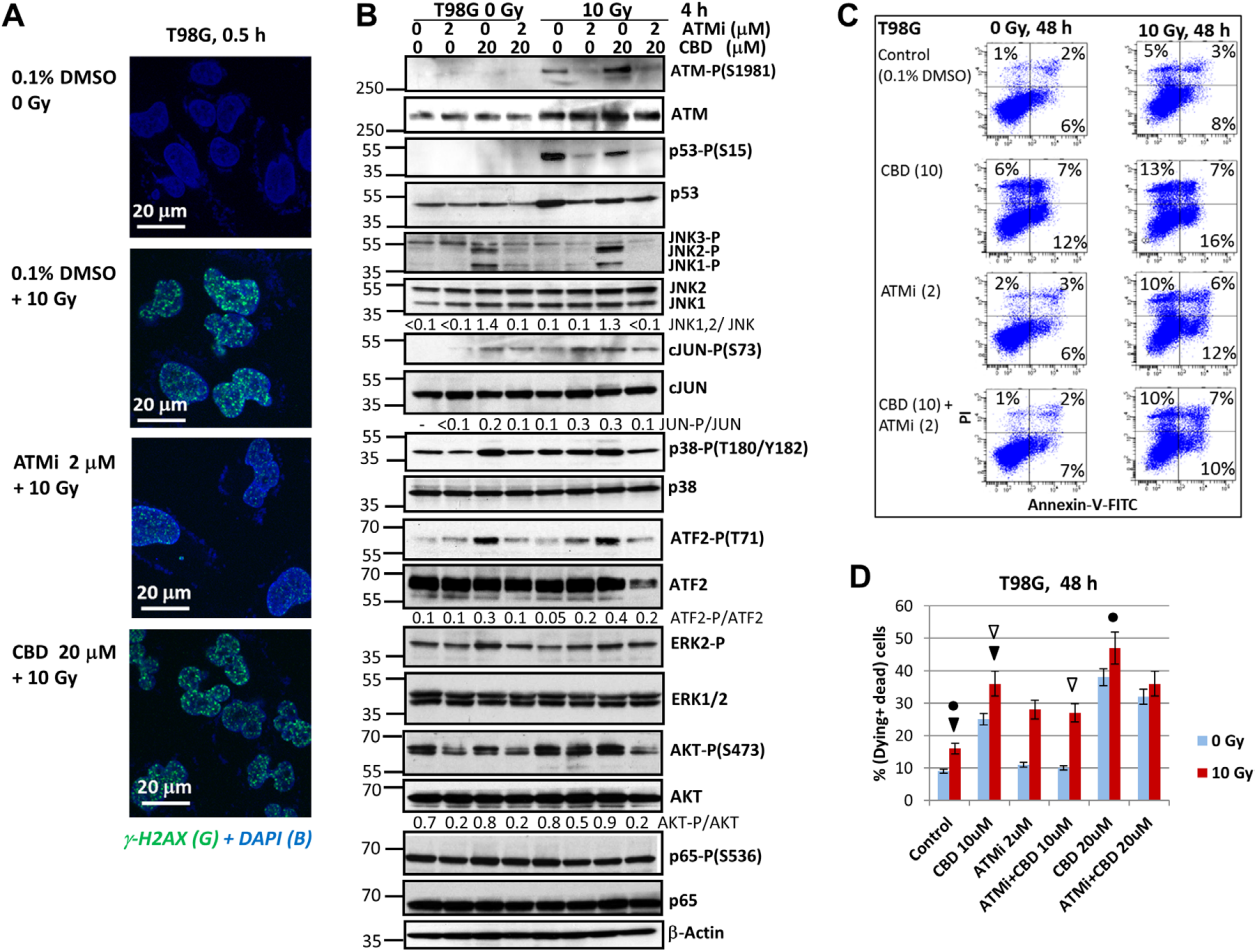
Changes in the pattern of cell signaling proteins of T98G human GBM cells and regulation of cell death after treatment with ATMi alone or in combination with CBD and γ-irradiation **(A)** DNA DSB were induced in the nuclei of treated T98G cells and detected by immunostaining with anti-γH2AX Ab (green foci) and DAPI staining of DNA (blue). **(B)** Western blot analysis of indicated signaling proteins from T98G cells was performed 4 h after treatments with CBD (10 μM), the ATMi KU60019 (2 μM), alone or in combination, without or with γ-irradiation (10 Gy). CBD, ATMi, and DMSO (a vehicle) were added to the cell cultures 30 min before irradiation. **(C and D)** Apoptotic levels in T98G cells were detected using Annexin-V-FITC and PI staining, as described in Figure 3A. A typical result **(C)** and the pooled results of four independent experiments are presented **(D)**. Error bars represent means ± S.D. (*p* < 0.05, Student's t-test). The arrows and circles indicate significant differences in cell death levels between control cells and cells after specified treatments.

Even though T98G GBM cells (*TP53mut; PTENmut*) are very different from U87MG GBM cells (*TP53wt; PTENmut*) in many respects, including the chromosome ploidy and sets of somatic mutations, there is a certain similarity in their response to CBD- and γ-irradiation treatment and the modulation on their cell signaling pathways after cotreatment with ATMi (Figure 5). Similarly to U87MG cells, γ-irradiation effectively induced γ-H2AX foci formation in the nuclei of T98G cells that was substantially blocked by ATMi (Figure 5A). Furthermore, ATMi prevented p53-(S15) phosphorylation and downregulated its protein levels in irradiated cells (Figure 5B). JNK activation by CBD in T98G cells was pronounced but somewhat decreased, compared to its activity in U87MG cells (see Figures 5B and 1B). A strong activation of MAPK p38 by CBD was also detectable in T98G cells followed by activation of ATF2 via Thr-71 phosphorylation, which could be suppressed in the presence of ATMi. Suppression of AKT1 activity in the presence of ATMi was also revealed. ERK2-P and NF-κB p65-P levels were relatively stable after treatment (Figure 5B). Numerous effects of ATMi on the crucial cell signaling pathways predict its modulating effects on cell growth and survival following treatment of T98 cells with CBD and γ-irradiation.

**Figure 6:**
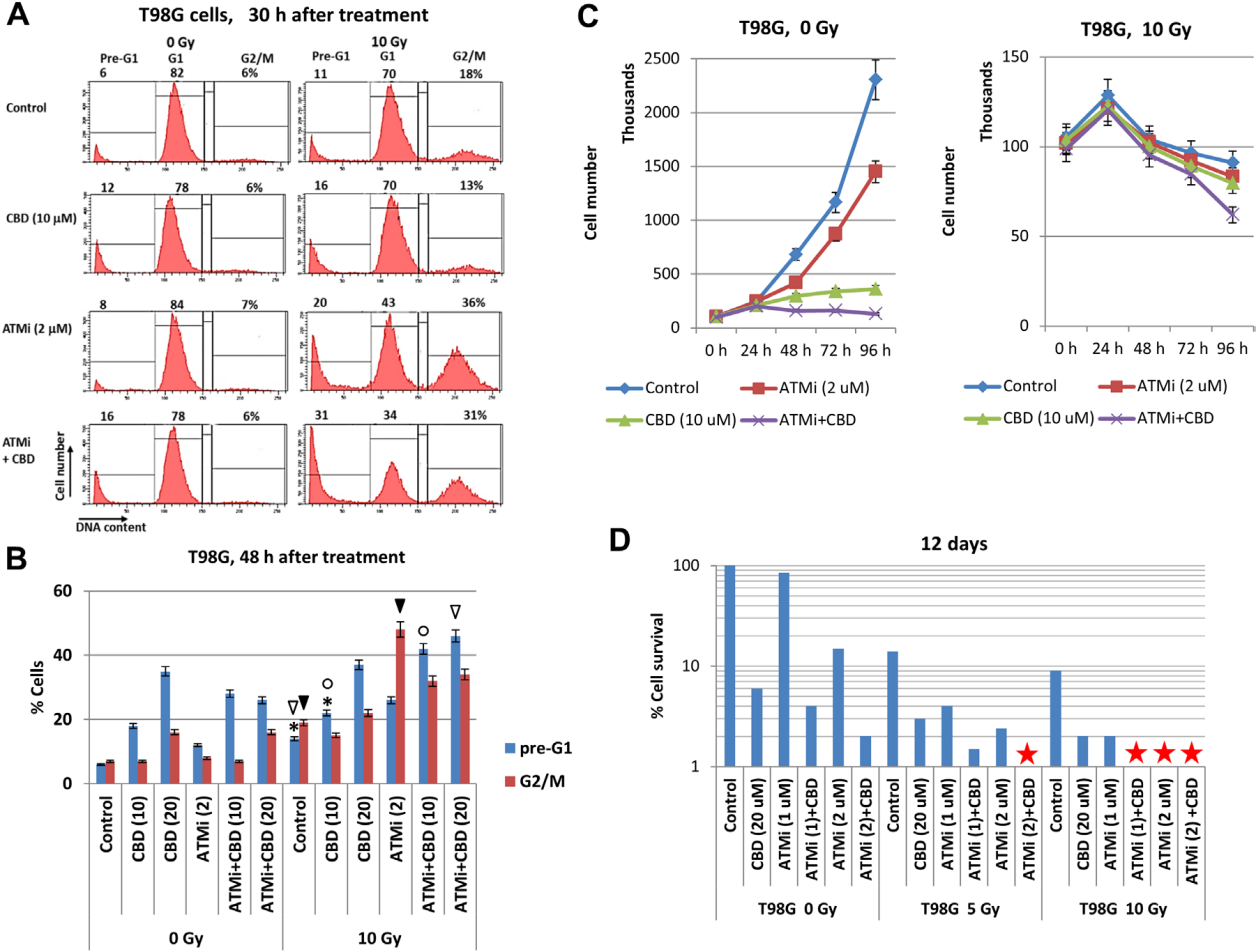
Cell cycle analysis, growth and clonogenic survival of T98G cells after treatments with indicated combinations of cannabidiol (CBD), the ATMi KU60019 and γ-irradiation (5-10 Gy) **(A and B)**. Cell cycle-apoptosis was performed 30-48 h after indicated treatments of T98MG cells with CBD (10 μM), ATMi (2 μM), and γ-radiation (10 Gy). CBD and ATMi, as well as a vehicle control (0.1% DMSO), were added 30 min before irradiation. Cells were stained with PI for determination of DNA content using the flow cytometry. A typical result is shown in the panel **(A)**. Pooled results of four independent experiments for T98G cells 48 h after treatments are shown in panel **(B)**. Error bars represent means ±S.D. (*p* < 0.05, Student's t-test). The stars, arrows, and circles indicate significant differences in the percentages of the pre-G1 or the G2/M cell populations after indicated treatments. **(C)** Kinetics of T98MG cell growth was monitored 0-96 h after indicated treatments: 0.1% DMSO (as control), ATMi (2 μM), CBD (10 μM) or a combination of ATMi+CBD without and with γ-irradiation. **(D)** Clonogenic survival assay was performed for cell cultures after indicated treatments with 0.1% DMSO (as control), ATMi (1-2 μM), CBD (20 μM) or a combination of ATMi+CBD followed by γ-irradiation. The final number of cell clones was determined after 12 days of cell culture growth. A ratio of a number of clones of treated cells to a number of clones of control cells reflected cell survival (percentage). Stars indicate the absence of survived clones.

The “gain of function” of mutated p53 resulted in additional resistance to temozolomide-induced apoptosis in T98G cells [32]. Nevertheless, (CBD+10Gy)-induced apoptosis was pronounced, while the presence of ATMi partially decreased pro-apoptotic and secondary necrotic levels induced by CBD at the 10-20 μM in non-irradiated and irradiated cells (Figure 5C and 5D). Surprisingly, the pre-G1 levels after treatment with ATMi+CBD+10Gy were actually increased in T98G cells, compared to treatment with CBD+10Gy, demonstrating the additive effect (Figure 6A and 6B). Inhibitory effects of combined treatments on cell proliferation were also highly detectable and especially strong for treatment with the triple combination of ATMi+CBD+10Gy (Figure 6C). For clonogenic survival, a combination of ATMi (2 μM), CBD (20 μM) and γ-irradiation (5 Gy) caused complete elimination of clones of GBM cells 12 days after treatment (Figure 6D). Furthermore, γ-irradiation at 10 Gy completely eliminated T98G clones pre-treated with [ATMi (1μM) + CBD (20μM)] or ATM (2μM), demonstrating higher efficiency of ATMi for radiosensitization of T98G cells.

The results of our study on U118MG cell death also demonstrated strong effects of γ-irradiation, ATMi, and CBD, alone or in combination, on upregulation of cell percentage at G2/M phase but moderate effects on levels of apoptosis (Supplementary Figure 1). Modulation of p53-P(S15) levels in non-irradiated and irradiated cells in the presence of ATMi was easily detectable (Supplementary Figure 1E). Paradoxically, this cell line demonstrated very low levels of JNK activation upon CBD treatment [21], while ERK1/2, MAPK p38, and AKT exhibited strong and stable activities (Supplementary Figure 1E). However, the triple combination of ATMi+CBD+10Gy moderately downregulated AKT1-P(S473) levels 4 h after treatment (Supplementary Figure 1E), with a substantial decrease of these levels at 24 h after treatment (not shown) allowing to partially suppress anti-apoptotic response. Finally, the clonogenic survival assay demonstrated the complete elimination of cancer cell clones 12 days after treatment with the triple combination of ATMi (2 μM), CBD (20 μM) and γ-irradiation at 5 Gy.

**Figure 7:**
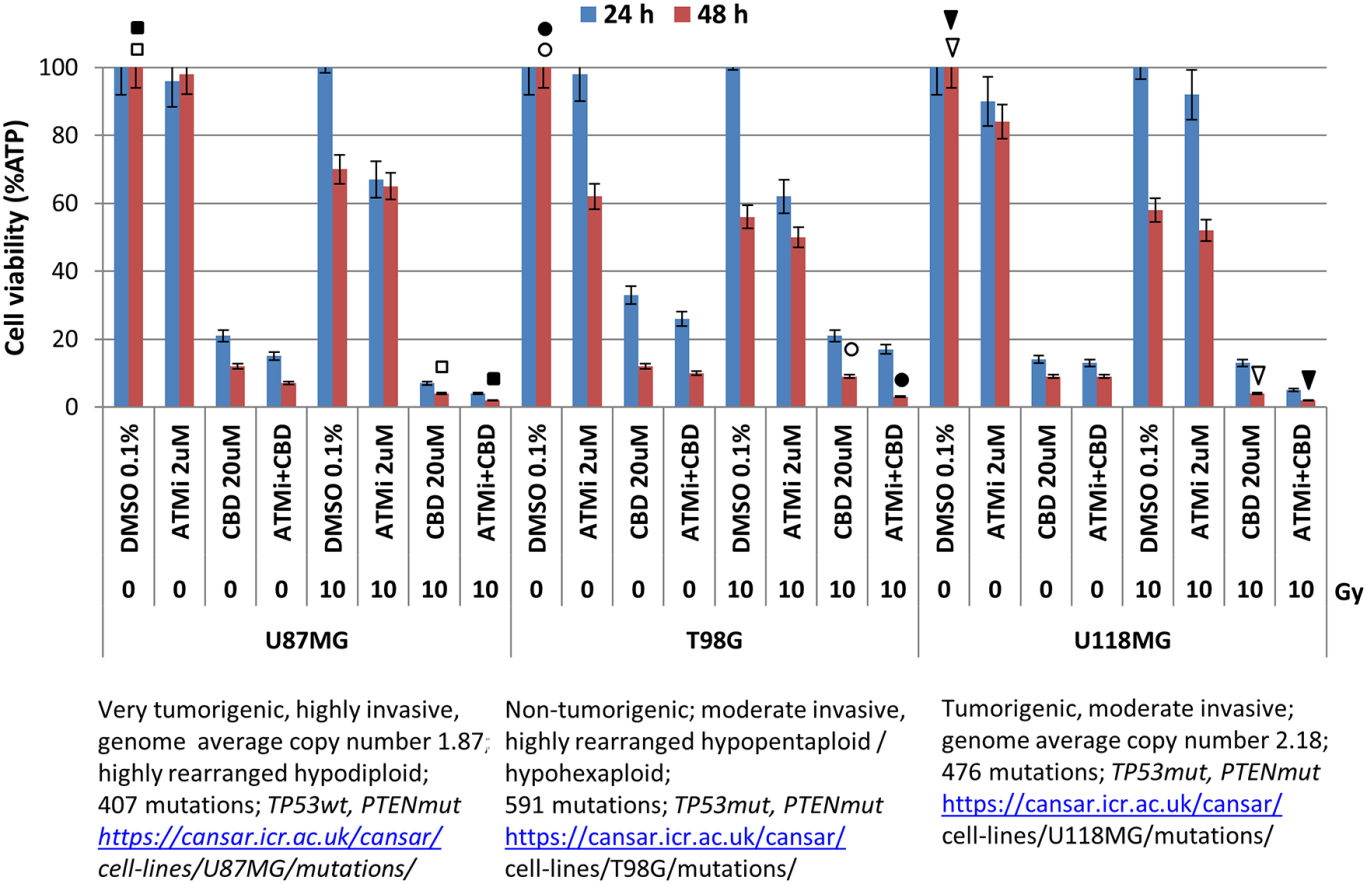
Effects of CBD (20 μM), ATMi (2 μM), and γ-irradiation (10 Gy), alone or in combination on GBM cell viability Cell viability assay (based on ATP-dependent luminescence intensity 24-48 h after indicated treatments) was performed for three glioblastoma lines after indicated treatments. Pooled results of four independent experiments 24-48 h after treatments are shown. Error bars represent means ±S.D. (*p* < 0.05, Student's t-test). Arrows, circles, and squares (open and black) indicate significant differences in cell viability after specified treatments.

Taken together, our data showed relatively similar features in upregulation of total cell death for three human GBM lines 12 days after treatment with a triple combination of killing factors, even though a role of apoptotic and non-apoptotic stimulation was different and cell-specific. The comprehensive comparison of cell viability for three GBM lines (using detection of ATP-dependent luciferase activity in CellTiter-Glo cell viability assay) further confirmed strong suppressive effects of the triple combination of 20 μM CBD, γ-irradiation at 10 Gy in and ATMi (2 μM) for the dramatic and almost complete elimination of GBM cells even 48 h after treatment (Figure 7). On the other hand, prolonged incubation of treated cells after the triple combination of CBD (20 μM), ATMi (2 μM) and a lower dose of γ-irradiation (5 Gy) also resulted in complete elimination of GBM clones 12 days after treatment as was observed in clonogenic survival assay (see Figures 4D, 6D and Supplementary Figure 1F).

### Secretory phenotypes of GBM cells after treatment by CBD, γ-irradiation, and ATMi: expression of pro-inflammatory cytokines

**Figure 8:**
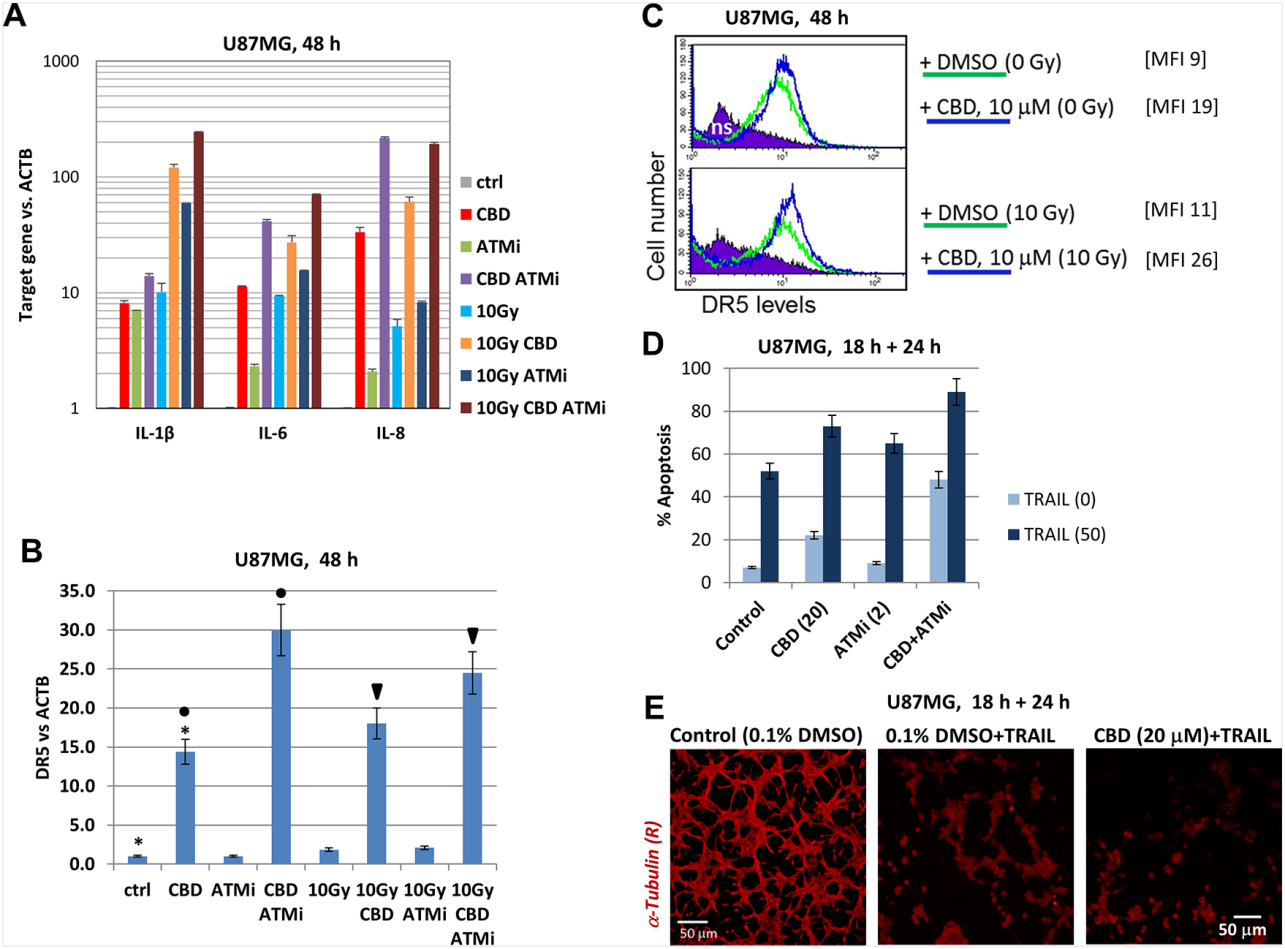
CBD-mediated control of pro-inflammatory cytokines and DR5/TRAIL-R2 in U87MG cells **(A and B)** Gene expression of pro-inflammatory cytokines (IL1β, IL6, and IL8), as well as TRAIL-R2 in U87MG cells, was determined by quantitative real-time PCR. mRNA was isolated 48 h after treatment with 0.1% DMSO (control vehicle), CBD (10 μM), ATMi (2 μM) and γ-irradiation (10 Gy) alone or in combination. The graphs indicate the fold change of target gene mRNA levels against time-point control after normalized to the reference gene (*beta-ACTIN*). The pooled results of four independent experiments are presented. Error bars represent means ± S.D. *(p* < 0.05, Student's t-test). **(C)** Effects of CBD (10 μM) and γ-irradiation (10 Gy) on surface expression of DR5/TRAIL-R2 were determined using immunostaining and FACS analysis. Median fluorescent intensity is shown in square brackets. **(D)** Effects of pretreatment (18 h) with CBD (20 μM), ATMi (2 μM) and their combination on apoptosis of U87MG cells induced by exogenous TRAIL (50 ng/ml) + cycloheximide (1 μg/ml). Apoptosis-cell cycle analysis of U87MG cells was performed and the percentage of pre-G1 (apoptotic) cells was determined using flow cytometry. The pooled results of three independent experiments are presented. Error bars represent means ± S.D. *(p* < 0.05, Student's t-test). **(E)** Microscopic view of cell death induced by exogenous TRAIL (50 ng/ml) or by pretreatment (18 h) of CBD (20 μM) followed by treatment with TRAIL (50 ng/ml for 24 h)

Endogenous and microenvironment-induced synthesis of pro-inflammatory cytokines is a characteristic feature of many different types of advanced cancers, including glioblastoma, especially after stress treatments [33]. In the current study, we addressed the regulation of transcription and protein expression of several pro-inflammatory cytokines (IL-1β, IL-6, and IL-8) by CBD, ATMi and γ-irradiation, alone or in combinations, in U87MG GBM cells (Figure 8) that could be critically important for *in vivo* conditions. Only modest changes in specific mRNA levels of these cytokines were observed at 4 h after treatment, while moderate changes were observed 24 h after treatment (data not shown). Finally, dramatic changes in transcription levels of the IL-1β, IL-6 and IL-8 mRNA occurred between 24 h and 48 h after the initiation of treatment by CBD alone or, especially, in combination with γ-irradiation (Figure 8A). ATMi in combination with (CBD+10Gy) additionally increased these levels for IL-1β, IL-6, and IL-8 48 h after treatment: the total normalized increase was at 200-fold for IL-1β and IL-8 while at 60-fold for IL-6, compared to the control levels (Figure 8A). In contrast, TGFβ1 levels were relatively stable (data not shown). Precise mechanisms that involved in dramatic upregulation of transcription of several cytokines by indicated combinations of treatment modalities need an additional investigation. However, strong inhibitory effects of small molecule inhibitors BMS344551 (IKK-NF-κB) and LY294002 (PI3K-AKT-NF-κB) on basal and (CBD + γ-irradiation)-induced levels of these cytokines (data not shown) highlights a critical role of permanently active NF-κB in regulating of basal expression of proinflammatory cytokines IL-1β, IL-6 and IL-8 [33].

Besides the indicated pro-inflammatory cytokines, the expression of the immunosuppressor FAS-L was detected in U87MG glioblastoma cells (Supplementary Figure 2A). Fas-L mRNA levels were strongly increased following CBD or CBD+ATMi treatment, likely due to JNK-cJUN/ATF2 upregulation since transcription factor AP1 (cJUN-ATF2) is a critical regulator of FAS-L expression [34] among other transcription factors. It was followed by a moderate CBD-mediated increase of FAS-L protein levels that was more pronounced in non-irradiated cells (Supplementary Figure 2B). Furthermore, the total protein and surface levels of FAS-receptor notably increased after irradiation (Supplementary Figure 2C), potentially allowing the paracrine/autocrine mechanism of FAS-L/FAS-mediated apoptosis in glioma cells. However, endogenous FAS-L-mediated apoptosis was not detected at pronounced levels in U87MG cells [21]. In contrast, FAS-L expression and secretion might be potentially important for immune surveillance in GBM [35]. CBD treatment did not notably affect permanently active NF-κB levels in glioblastoma cells (Figures 1C and 5B).

Another signaling pathway that could play an essential role in GBM cell death under stressful conditions is TRAIL-TRAIL-R2 (21). The endogenous expression of TRAIL in both U87MG and U118MG GBM lines was low and only modestly increased after irradiation (data not shown). An alternative possibility for increasing TRAIL-induced apoptosis in GBM cells was CBD-induced upregulation of gene, protein and cell surface expression of TRAIL-R2/DR5 (Figure 8B and 8C) and use of the exogenous TRAIL for induction of apoptosis. Indeed, pre-treatment with CBD, ATMi or combination of CBD+ATM for 18 h followed by treatment with the exogenous TRAIL (50 ng/ml) resulted in high levels of TRAIL-mediated apoptosis after subsequent 24 h of incubation (Figure 8C and 8D).

### PD-L1 expression in U87MG cells following CBD treatment and γ-irradiation

A strategy of glioma gene- and epigenetic regulation directed by the Darwinian clonal selection during carcinogenesis is tightly linked with the acquisition of immunosuppressive functions of cancer cells, including boosted FAS-L, PD-L1 and PD-L2 expression, their proper posttranslational modifications and protein trafficking to the cell surface. Beside FAS-L/FAS, the interaction of PD-L1 of cancer cells with the PD1 receptor on the surface of CD8^+^ T-lymphocytes is a critical reaction of the immune checkpoint resulting in functional loss of anti-cancer activity of CD8^+^ T-lymphocytes [36].

**Figure 9:**
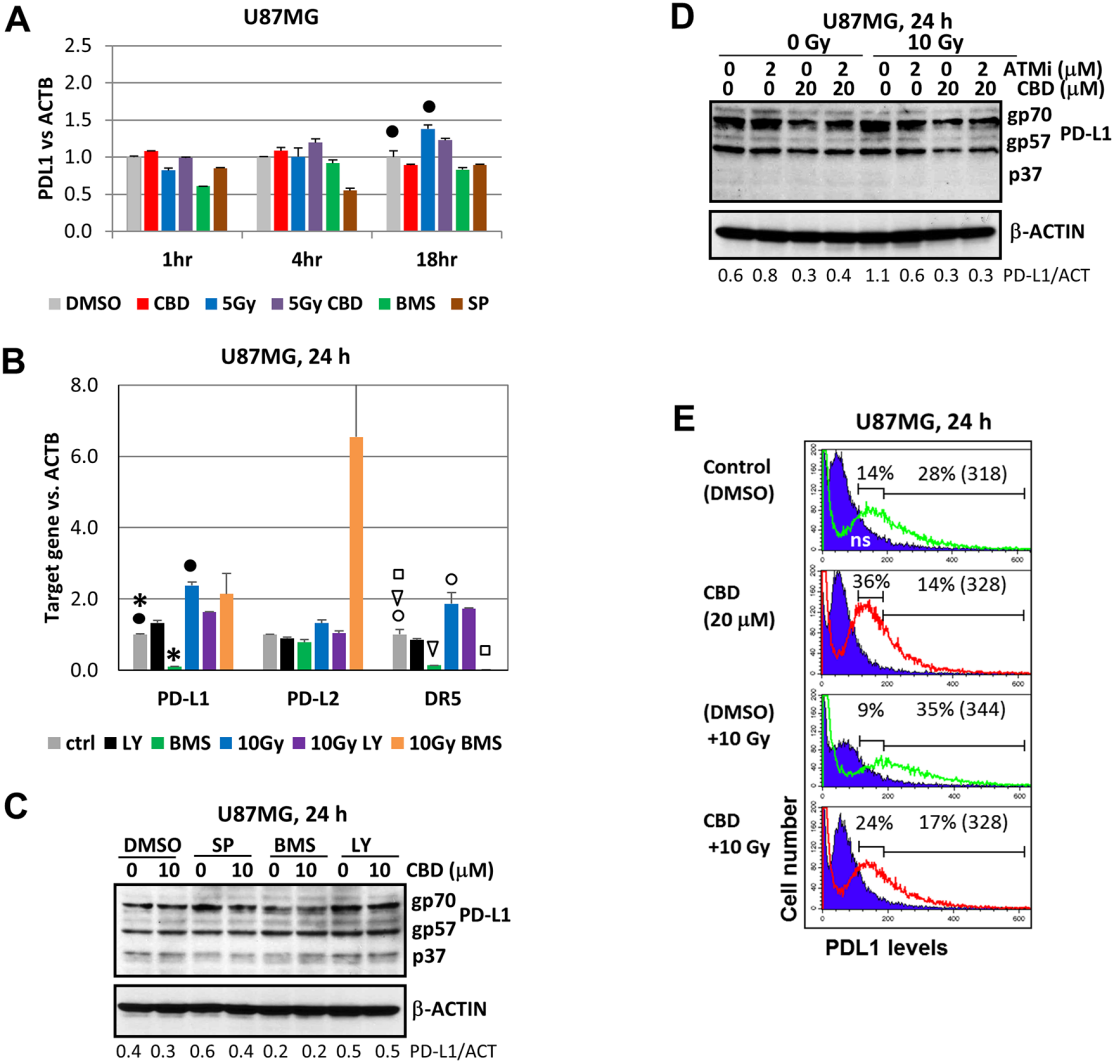
Gene and protein expression and surface levels of PD-L1 after treatment with CBD, alone or in combinations **(A and B)** The quantitative real-time PCR analysis of the PD-L1, PD-L2 and DR5 mRNA levels 1-24 h after treatments of U87MG by CBD (10 μM), BMS345541 (20 μM), LY294002 (40 μM) and γ-irradiation (5-10 Gy), alone or in combination. The graphs indicate the fold change of target gene mRNA levels against time-point control after normalized to the reference gene (*beta-ACTIN*). The pooled results of four independent experiments are presented. Error bars represent means ± S.D. *(p* < 0.05, Student's t-test). Black stars, black and open circles, arrows and squares indicate a significant difference in mRNA levels between control and specifically treated cells. **(C and D)** Western blot analysis of PD-L1 protein expression after CBD (10-20 μM) treatment, alone or in combination with SP600125 (20 μM), BMS345541 (20 μM), LY294002 (40 μM), ATMi (2 μM) and γ-irradiation (10 Gy) in U87MG cells. **(E)** Negative effects of CBD on PD-L1 surface expression in non-irradiated and irradiated (10 Gy) cells were determined using immunostaining and the flow cytometry. Percentage of gated cells (with high and low expression) is indicated. Median fluorescent intensity is indicated in the brackets.

We observed that CBD alone did not notably affect *PDL1* transcription in U87MG cells (1-24 h after treatment), while γ-irradiation alone or in combination with CBD only slightly increased *PDL1* mRNA levels (Figure 9A). BMS345541, a small molecule inhibitor of IKK-NF-κB marginally downregulated *PDL1* transcription 1-18 h after treatment and exhibited a variable inhibitory effect for the basal *PD-L1* transcription (Figure 9A and 9B). Interestingly, BMS345541 substantially increased radiation-induced *PD-L2* expression. In contrast, BMS345541 suppressed both the basal and radiation-induced levels of *DR5/TRAIL-R2* that was used as a control target (Figure 9B). Three PD-L1 protein species with the apparent molecular weight of 33-37, 55-57 and 68-70 kD were revealed in U87MG cells (Figure 9C). The major species, gp70 and gp57, were stable glycoproteins localized on the plasma membrane [37]; the minor non-modified p37 subunit was hardly detectable (or sometimes undetectable) in glioma cells (Figure 9C and 9D). Pretreatment with a small molecule inhibitor BMS345541 (but not SP600125 and LY294002) modestly decreased levels of gp70 (Figure 9C). In contrast, CBD treatment alone or in combination with γ-radiation notably decreased protein levels of gp70 and gp57 (Figure 9C), likely through modulation of their stability by CBD-activated kinases linked with the proteasome system [37]. Levels of surface expression of PD-L1 were also under negative control of CBD: immunostaining and the flow cytometry demonstrated increased levels of PD-L1 surface expression after γ-irradiation, but decreased level after treatment with CBD alone or in combination with radiation 24 h after treatment (see a decrease of the percentage of cells with high surface expression of PD-L1 on Figure 9E).

Taken together, our results show pronounced but mild negative effects of CBD on PD-L1 gene/protein/cell surface expression, in sharp contrast to CBD-mediated positive effects on DR5/TRAIL-R2 expression (see Figure 8), highlighting the critical features of an anti-cancer strategy driven by CBD.

### Effects of CBD, alone or in combination with γ-irradiation and ATMi on the viability of U87MG neurospheres

**Figure 10:**
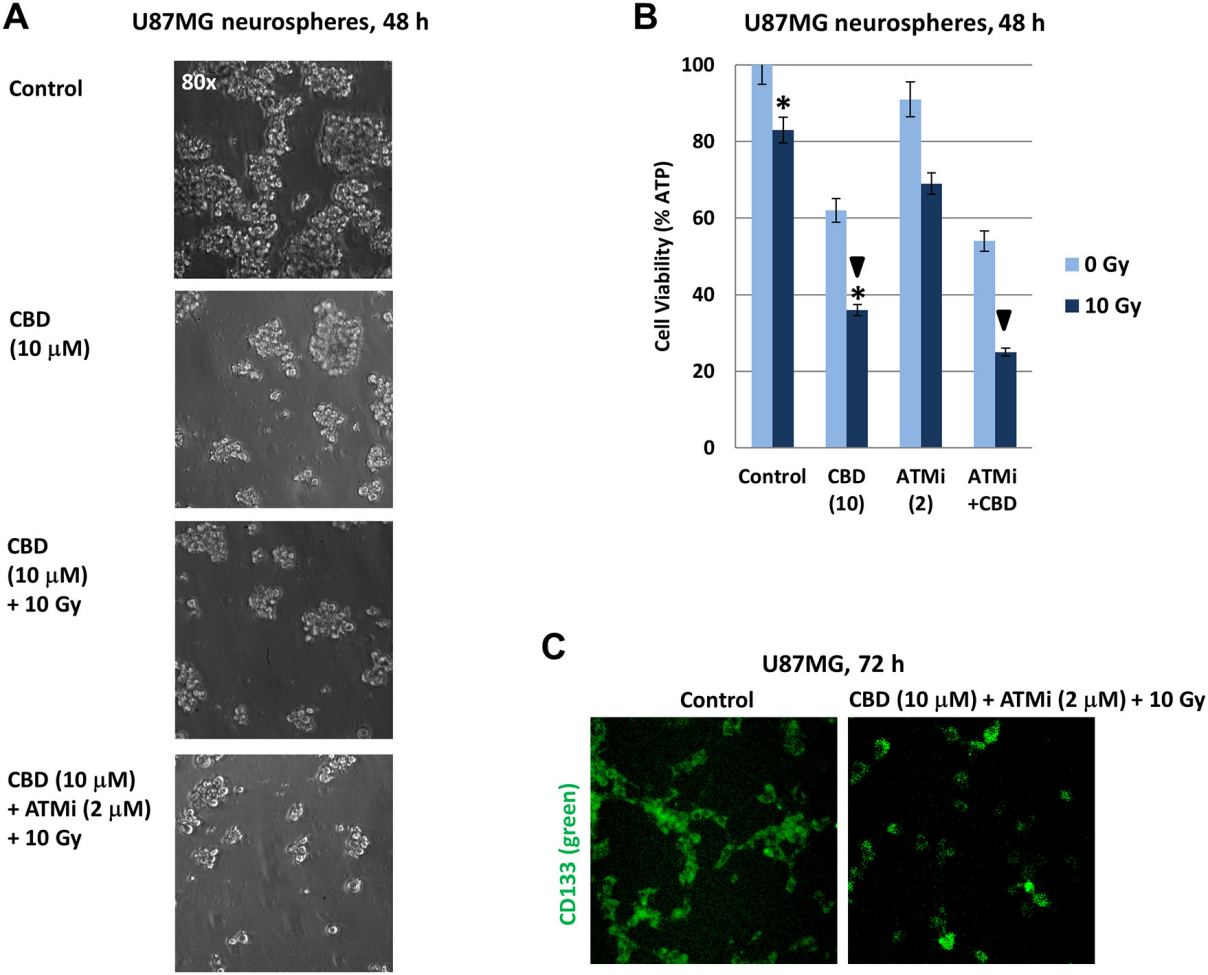
Induction of cell death in U87MG spheroid cultures following treatments with CBD, ATM inhibitor (ATMi), and γ-irradiation (10 Gy), alone or in combination **(A)** Phase-contrast images of U87MG neurospheres 48 h after treatment with 0.1% DMSO (control) or CBD (10 μM), alone or in combination with γ-irradiation (10 Gy) or γ-irradiation + ATMi (2 μM). **(B)** Cell viability assay was performed for U87MG neurosphere cultures 48 h after indicated treatments. Pooled results of four independent experiments 48 h after treatments are shown. Error bars represent means ±S.D. (*p* < 0.05, Student's t-test). Arrows and stars indicate significant differences in cell viability after specified treatments. **(C)** U87MG neurospheres from suspension cultures (72 h after treatment) were attached to coverslips and immunostained with anti-CD133 mAb (green).

In spite of a restricted similarity between primary GBM cells *in vivo* and established cell lines after many passages in culture conditions (such as U87MG, U118MG and T98G glioblastoma lines), a recent comprehensive study highlighted the importance of established cell lines that represent relatively similar patterns of gene alteration as cancer cells *in vivo* [38]. On the other hand, glioma spheroid cultures are considered to be a source of glioma stem-like cells (glioma-initiating cells) that are especially resistant to treatment, including γ-irradiation [39, 40]. In general, patient-derived glioma stem-like cells grown in neural stem cell culture as neurospheres may retain most of genomic and epigenetic characteristics of primary patient tumors, thus representing an additional model system in glioma investigation [41]. U87MG spheroid culture enriched by CD133^+^ glioma stem-like cells was established as described in “Materials and Methods” and in our previous publication [42]. Increasing complexity of treatment using CBD (10 μM), [CBD (10 μM) + 10 Gy] and [CBD (10 μM) + 10 Gy + ATMi (2 μM)] resulted in the progressive decline of viability of U87MG neurospheres, revealed by several different approaches: phase-contrast microscopy, cell viability assay and immunostaining with anti-CD133 mAb (Figure 10A-10C). Even the viability of U87MG neurospheres after treatment was higher compared to the corresponding adherent culture, providing data on the sensitivity of glioma neurospheres to CBD, alone or in combination, which look promising for suppression of cancer growth. These observations that extended recently published data on treatment of U87MG glioma-initiating cells by a combination of CBD, THC and temozolomide [43] still required additional studies for confirmation of the efficacy of a used combination through treatments *in vivo*.

### CBD-mediated effects on human TS543 proneural glioma cells

**Figure 11:**
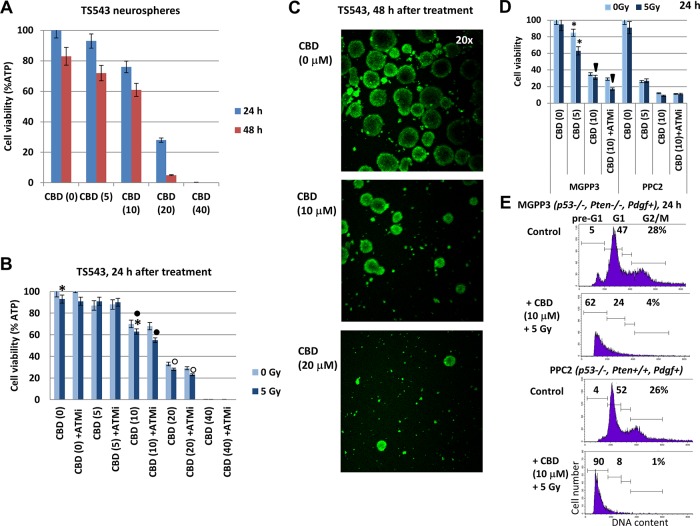
The viability of TS543 human glioma spheroid cultures following treatments with CBD, ATM inhibitor (ATMi), and γ-irradiation (5 Gy), alone or in combination **(A)** Dose-dependent response of TS543 neurospheres to CBD (0-40 μM). **(B)** Combined treatment of neurospheres using CBD, γ-irradiation (5 Gy) and ATMi (2 μM). Cell viability assay was performed for TS543 neurosphere cultures 24-48 h after indicated treatments. Pooled results of four independent experiments 24-48 h after treatments are shown. Error bars represent means ±S.D. (*p* < 0.05, Student's t-test). Stars and squares indicate significant differences in cell viability after specified treatments. **(C)** CBD-treated (48 h) and control TS543 neurospheres were additionally incubated with CitoTrucker green for 30 min and analyzed using fluorescent microscopy. **(D)** Effects of CBD (5-10 μM), ATMi (2 μM) and γ-irradiation (5 Gy), alone or in combination, on the viability of mouse MGPP3 and PPC2 glioma neurosphere cultures 24 h after treatment. Pooled results of four independent experiments are shown. Error bars represent means ±S.D. (*p* < 0.05, Student's t-test). Arrows and stars indicate significant differences in cell viability after specified treatments. **(E)** Effects of CBD (10 μM) and γ-irradiation (5 Gy) on pre-G1 (apoptotic) levels in MGPP3 and PPC2 glioma cells (adherent culture) 24 h after treatment. Cells were pretreated with 0.1% DMSO (control) or CBD 30 min before irradiation. The nuclei of control and treated cells were stained with PI and DNA content in cells was determined using flow cytometry.

Further progress of our study was linked with an additional investigation using combined treatment on the human TS543 (*PDGF+*) proneural glioma cell line at a low passage, which also could be grown either as neurospheres (Figure 11) or adherent cells. TS543 neurospheres were extremely sensitive to CBD, demonstrating dose-dependent response (Figure 11A) 24-48 h after treatment and resulting in a complete elimination of live cells after treatment with 40 μM CBD (Figure 11B). Additional 30-min incubation of control and CBD-treated cells (48 h) with CellTracker green (#C7025 from “Invitrogen”) and confocal microscopy also revealed CBD-mediated damage of neurospheres and a strong downregulation of cell viability (Figure 11C). CBD-mediated radiosensitization, especially in the presence of ATMi, was detected for intermediate levels of CBD (10-20 μM) (Figure 11B).

To further extend our observation on CBD-mediated death for proneural glioma cultures at a low passage, we took advantage of two mouse glioma lines created in the laboratory of Dr. P. Canoll (Columbia University, New York): PPG2 *(Pdgf+, p53−/−, Pten+/+)* and MGPP3 *(Pdgf+, p53−/−, Pten−/−).* MGPP3 represents a mouse proneural glioma cell line [44, 45]. Both cell cultures could grow either as neurospheres in suspension culture or as adherent cells in laminin-treated plates. One of the critical targets of CBD in human and mouse GBM used in the current study was the PI3K-AKT pathway (see Figures 1B and 5B). A series of experiments were performed with mouse glioma cells grown as neurospheres (Figure 11D). Due to *Pten* knock-out and the subsequent PI3K-AKT over-activation in MGPP3 cells, there was a substantial decrease in sensitivity to CBD, alone or in combination with γ-irradiation for this line, compared to PPG2 (Figure 11D). Pretreatment with ATMi (2 μM) and γ-irradiation (5 Gy) that targeted numerous signaling pathways, including PI3K-AKT, further decreased the survival of the CBD-treated MGPP3 neurosphere culture. Additionally, significant levels of CBD-induced apoptotic death (as % cells in pre-G1 area) were revealed in MGPP3 adherent cell culture 24 h after treatment. As expected, the corresponding death levels in PPC2 cells were higher (Figure 11E).

Taken together, our results demonstrated the ability of CBD to partially overcome GBM resistance to treatment, even in the case of PI3K-AKT over-activation, which was a critical component of this resistance pathway [46].

## DISCUSSION

Glioblastoma (GBM) is a hallmark of cancer resistance to treatment, including radioresistance and cancer-associated immunosuppression. Despite aggressive therapies, the prognosis for GBM patients remains dismal. Identification of novel pharmacological targets and development of new therapeutic strategies are urgently needed. There are several potentially efficient approaches among others used for GBM trials to overcome, at least partially, extremely damaging effects of cranial irradiation and chemotherapy and substantially increase a selectivity for killing of brain cancer cells: 1) the precise external beam radiation therapy by Gamma Knife radiosurgery that allows minimization of radiation-induced necrosis to surrounding brain tissue [47]; 2) application of small molecule inhibitors of the specific signaling pathways, which are overactive in the particular glioblastoma type; and 3) advanced immunotherapy of cancer [48, 49].

A complementary possibility for advanced GBM treatment is the activation of cell signaling receptors, which could regulate gene expression controlling cell death specifically in cancer cells rather than in non-malignant cells. Cannabinoid receptor CB1 is the most abundant cell surface receptor in the brain; cannabinoid receptor CB2 is also widely expressed in different types of cells, including both normal glial cells and GBM [50]. Both receptors, by binding endocannabinoids, activate signaling cascades, which control proliferation, survival and other normal functions. Dysregulation of cannabinoid-mediated signaling that tightly linked with the COX2-dependent production of prostaglandin-E2 is one of the leading strategies of the clonal selection during glioma carcinogenesis [51]. Paradoxically, it also provides a possibility for induction of GBM cell death by exogenous cannabinoid treatment. Numerous investigations in the last decade demonstrated cytotoxic effects of cannabinoids on human and mouse glioblastoma cells expressing both CB1 and CB2 receptors [12, 16, 19, 52, 53]. However, a specificity of the signaling mechanisms involved in the regulation of GBM cell death by cannabinoids is still not completely investigated. In previously published studies, we and others elucidated the effects and probable mechanisms of radiosensitization of GBM cells using cannabinoids [19–21].

From the numerous members of the cannabinoid family, we were focused on actions of cannabidiol (CBD) without psychogenic activity, alone or in combination with γ-irradiation, establishing a promising combination for induction of cell death via apoptosis or necrosis in human GBM cells. The main goals of our current study were: 1) further increase efficiency of killing of human GBM cells by combined treatment of CDB and γ-irradiation using additional suppression of ATM kinase activity; 2) elucidate a relative input of apoptotic and necrotic mechanisms following treatment of GBM cells with a triple combination of CBD, ATMi, and γ-irradiation; 3) investigate effects of γ-radiation and CBD, alone or in combination, on the regulation of DR5/TRAIL-R2 expression in glioma cells; 4) elucidate signaling pathways and their corresponding targets, which control the expression of *PDL1* in glioma cells before and after treatment with CBD and γ-irradiation. Such data could be critical for the subsequent *in vivo* animal experiments.

Based on the results of the present study, we were able to further increase cytotoxic effects of combined treatment of CBD at intermediate (10-20 μM) doses and γ-irradiation (5-10 Gy) by inhibition of ATM kinase activity using a small molecule inhibitor KU60019. It caused a relatively minor downregulation of (CBD + γ-irradiation)-induced apoptosis simultaneously with a strong upregulation of a ratio of the G2/M-arrested glioma cells that finally blocked glioma cell proliferation. Two recent publications also described the strong negative effects of new ATM kinase inhibitors combined with γ-irradiation on glioma cells and GBM development [54, 55].

The mechanism of natural surveillance and killing of cancer cells based on the continuous maintenance of the strong and specific immune response has been successfully used in the recent strategy of immunotherapy, due to the introduction of immune checkpoint inhibitors, including inhibitory antibodies to PD1 or its ligand PD-L1, which is often overexpressed by cancer cells [56–58]. Unfortunately, this variant of immunotherapy successfully works only in six types of immunologically “hot tumors”: bladder cancer, head and neck cancers, kidney cancer, liver cancer, melanoma, and non-small cell lung cancer. The challenging problem is to apply immunotherapy for cancers that are immunologically “cold”, such as glioblastomas, ovarian, prostate, and pancreatic tumors [36].

There are, however, significant problems in the application of immunotherapy for brain tumor treatment, such as the blood-brain barrier and multiple well-pronounced immunosuppressive mechanisms, including FAS-Ligand expression by GBM that could induce FAS-L/FAS-mediated apoptosis of immune lymphocytes in the brain [35], as well as triggering T-lymphocyte checkpoints by PD-L1 surface expression in GBM cells and an additional permanent expression of immunosuppressors TGFβ1 and TGFβ2 by GBM cells [59, 60]. Interestingly, we observed dramatic upregulation of FAS-L expression in U87MG glioblastoma cells during the apoptotic commitment of these cells induced by CBD (see Supplementary Figure 2A and 2B), even the role of FAS-L/FAS-mediated suicide of GBM cells was not critical, due to a low endogenous expression of FAS Receptor [36].

One of the critical questions linked with a possible application of CBD or CBD+ATMi as anti-glioma agents was about their modulating effects on *PDL1* gene-, protein- and surface expression. The classical pathway that activates *PDL1* gene expression is mediated by interferon-γ produced and secreted by natural killers and CD8^+^ T-lymphocytes [36]. Since the *PDL1* promoter contains four potential NF-κB-binding sites, two STAT3-binding sites and potential AP1- and NF-AT-binding sites, a role of EGFR kinase activation, as well as IKK-NF-κB, AKT-NF-κB, JAK2-STAT3 and MAPK-AP1 activating pathways in the regulation of *PDL1* gene expression was highlighted [61–65]. Furthermore, a role of radiotherapy in the upregulation of PD-L1 expression was also established [65, 66]. Our observations revealed relatively minor negative effects on CBD and γ-irradiation PD-L1 gene- and protein expression in U87MG GBM cells, demonstrating an additional advantage of CBD treatment for this type of cancer.

In contrast, substantial upregulation of gene, protein and surface expression of DR5/TRAIL-R2 after CBD treatment, alone or in combination with irradiation, further sensitize GBM cells to the external TRAIL-induced apoptosis (see Figure 8). Recent attempts to use neural stem/progenitor cells, which express TRAIL, as vehicles to kill TRAIL-R2-positive GBM cells demonstrated promising results [67]. On the other hand, pre-clinical testing imipridone ONC201 for cancer treatment revealed several similar mechanisms with CBD-mediated induction of cell death [68, 69], such as targeting mitochondria and AKT/ERK inactivation linked with TRAIL/TRAIL-R-mediated apoptosis.

Furthermore, we observed the enhanced secretory phenotype of cancer cells with massive production of pro-inflammatory cytokines, IL1β, IL6 and IL8, as well as death ligands, FAS-L and TRAIL, after combined treatment of GBM cells. Besides its general pro-inflammatory function, IL6, which is a widely-distributed cytokine in the nervous system, is critically important for glial differentiation of normal neural precursor cells and adult glioma differentiation of glioma stem-like cells [70, 71]. Potentially, CBD-induced activation of IL6 expression in stress conditions might promote the viability and differentiation of a small population of glioma stem-like cells that were resistant to killing agents used [72]. We also observed a protective effect of IL6 for (CBD + 5Gy)-induced apoptosis in the whole population of U118MG cells [21].

However, the challenging question regarding local and systemic effects of CBD alone or in combination with γ-irradiation on the immune system needs additional investigations, since CBD is also known as an inducer of cell death of immune cells and modulator of cytokine production in monocytes [73, 74]. On the other hand, the killing of tumor-infiltrating macrophages by CBD appears a promising therapeutic strategy in advanced tumors where macrophages exhibit protumorigenic functions [73, 75].

Clinical trials with cannabinoids for GBM treatment have been very restricted, even providing some optimistic preliminary results [2, 76, 77]. Unfortunately, there are no available data from large, placebo-controlled trials regarding the systemic anti-cancer effect of cannabinoids. The first clinical pilot study published several years ago demonstrated that intracranially administered THC was safe in recurrent GBM patients [78]. A recently publicized phase II, a placebo-controlled clinical trial with recurrent GBM patients, provides promising data for the potential efficacy of cannabinoids as adjuvant anti-cancer drugs. In this study, 12 patients were randomized to a combination of THC and CBD in addition to dose-intensive temozolomide, whereas the remaining 9 patients were randomized to placebo plus temozolomide. The proof of concept study showed a significantly higher one-year survival rate in the cannabinoid group (83% versus 53% in the placebo cohort). Furthermore, the median survival for the cannabinoid group was greater than 550 days compared with 369 days in the group randomized to placebo (GW Pharmaceuticals 2017 press release; https://www.clinicaltrials.gov/ Identifiers: NCT01812616, NCT01812603). Several ongoing clinical trials for newly-diagnosed GBM patients are studying the role of cannabinoids and results are pending (https://www.clinicaltrials.gov/ Identifiers: NCT03529448, NCT 03246113).

In the present study, we used combined treatment of CBD, as an apoptotic inducer in GBM, and γ-radiation together with the ATM inhibitor KU60019 for induction of irreversible G2/M arrest accompanied by necrosis. In summary, we conclude that radiation-induced death of several human and mouse GBM lines in culture conditions (including cultures of neurospheres) could be substantially enhanced by CBD-mediated signaling in concert with ATM-kinase inhibition, via both apoptotic and non-apoptotic death pathways. We observed these death-inducing effects not only in the “old” classical cultures of human GBM (U87MG, U118MG and T98G) but also in more recently established human TS543 and mouse proneural glioma lines at a low passage. Potentially, these data could improve the therapeutic ratio of GBM and partially overcome cancer therapy-induced severe adverse side effects.

## MATERIALS AND METHODS

### Reagents

ATM kinase inhibitor (ATMi) KU60019, PI3K inhibitor LY294002, IKK inhibitor BMS345541, MAPK p38 inhibitor SB203580 and JNK inhibitor SP600125 were purchased from Calbiochem (La Jolla, CA, USA). Human soluble Enzo Killer-TRAIL (recombinant #ALX-201-073-C020) and anti-human TRAIL Ab (#ALX-210-732) were purchased from Enzo Life Sciences (San Diego, CA, USA). Cannabidiol (exempt preparation; #90081) was obtained from Cayman Chemical (Ann Arbor, MI).

### Glioma cell lines

Three lines of human glioblastoma (GBM) were obtained from the ATCC: i) U87MG (HTB-14): very tumorigenic, highly invasive, highly rearranged hypodiploid with several hundred mutations, *TP53wt, PTENmut;* ii) U118MG (HTB-15): tumorigenic, moderate invasive; several hundred mutations, *TP53mut, PTENmut;* iii) T98G (CRL-1690): non-tumorigenic; moderate invasive, highly rearranged hypopentaploid/hypohexaploid, several hundred mutations, *TP53mut, PTENmut* [79, 80]. There are some contradictions about the origin and maintenance of the U87MG (HTB-14) glioma cell line in the ATCC [81]; however, they do not interfere with the results of the present study. All GBM lines were cultured as previously described in DMEM with 10% FBS and 1% pyruvate [82]. For neurosphere formation, U87MG glioblastoma cells were cultured in the serum-free media DMEM/F12 supplemented with 2 mM GlutaMAX, bFGF (20 ng/ml), EGF (20 ng/ml) and B27 supplement (2%). All reagents were obtained from Gibco/Life Technologies (Carlsbad, CA, USA). After 15-20 passages, U87MG neurosphere culture was significantly enriched by CD133^+^ cells. Additionally, human TS543 proneural glioma cell line at low passage was grown as neurosphere culture [83] using *in vitro* proliferation kit for human neural stem cells NeuroCult NS-A (StemCell Technology, #05751) supplemented with heparin (2 μg/ml), human bFGF (10 ng/ml) and human EGF (20 ng/ml). Mouse glioma lines PPG2 *(Pdgf+, p53−/−, Pten+/+)* and MGPP3 *(Pdgf+, p53−/−, Pten−/−)* were generated by Dr. Peter D. Canoll (Columbia University, New York, NY). These cells were cultured as previously described using the protocol from Dr. Peter Canoll laboratory [44, 45].

### Immunocytochemistry analysis

To label active mitochondria, live glioblastoma cells were incubated with MitoTracker Green FM (Molecular Probes #M7514) using on Invitrogen Molecular Probes protocol. Cells were fixed with 4% paraformaldehyde in PBS for 30 min. Then immunochemical staining was performed using standard protocols. Glioblastoma cells were stained with α-Tubulin (mAb #T5168 from Sigma) and phospho-Histone H2A.X Ser139 (mAb #05-636 from Millipore), as well with anti-CD133 (mAb #64326 from Cell Signaling Technology). The secondary Abs were Alexa Fluor 594 goat anti-mouse IgG from Molecular Probes/Life Technologies (Carlsbad, CA, USA). A Nikon A1 scanning confocal microscope on an Eclipse TiE microscope stand (Nikon Instruments, Melville, NY) was used for immunofluorescence image acquisition (as Z-series projection) and analysis. Images were further analyzed and quantified using ImageJ software (NIH).

### Irradiation procedures

To determine sensitivity to γ-irradiation, plated cells were exposed to radiation from a Gammacell 40 ^137^Cs irradiator (dose rate, 0.82 Gy/min) at Columbia University. Six to 48 h after irradiation, cells were stained with PI or Annexin-V-FITC + PI and analyzed by flow cytometry for cell cycle-apoptosis studies.

### FACS Analysis of TRAIL-R2/DR5, FAS and PD-L1 levels

Surface levels of TRAIL-R2/DR5 (PE anti-human DR5 Ab, eBioscience #12-9908), FAS/CD95 (PE anti-human CD95 mAb, BD Pharmingen #555674), PD-L1 (CD274) (PE anti-human mAb eBioscience #12-5983) on human GBM cell lines were determined by staining with the corresponding Abs and subsequent flow cytometry. A FACS Calibur flow cytometer (Becton Dickinson, Mountain View, CA, USA) and the CellQuest program were used to perform flow cytometric analysis. All experiments were independently repeated 3-5 times.

### Cell viability and cell death studies

The CellTiter-Glo Luminescent Cell Viability Assay (“Promega”, Madison, WI53711, USA; #G7570) based on quantitation of ATP due to ATP-dependent luminescent signal, was performed using the manufactory's protocol. For the induction of apoptosis, cells were exposed to CBD (5-40 μM), KU60019 (1-5 μM) and γ-irradiation (5-10 Gy) alone or in combination. In some experiments, small molecule inhibitors of cell signaling pathways were additionally used. Furthermore, apoptosis was induced TRAIL and CHX alone or in combination. Apoptosis levels (% of apoptotic nuclei) in cells after fixation and permeabilization by 70% ethanol were assessed by propidium iodide (PI) staining and quantifying the percentage of hypodiploid nuclei (pre-G1) using FACS analysis. Alternatively, staining of fresh cells by Annexin-V-FITC + PI and quantifying the percentage of Annexin-V-FITC-positive, PI-negative cells (early apoptotic), Annexin-V-FITC-positive, PI-positive cells (late apoptotic) and Annexin-V-FITC-negative, PI-positive cells (secondary necrotic) was performed using reagents from BD Pharmingen (San Diego, CA) that was followed by the flow cytometry on FACS Calibur flow cytometer (Becton Dickinson) using the CellQuest program or on BD FACS Canto System from BD Biosciences. Clonogenic survival assay of glioblastoma cells was performed after treatment with increased doses of CBD and γ-radiation in the presence or absence of an ATM inhibitor KU60019 (1-2 μM) using a standard method.

### Western blot analysis

Total cell lysates (50 μg protein) were resolved on SDS-PAGE, and processed according to standard protocols. The antibodies used for Western blotting included anti-β-Actin mouse mAb (Sigma, St. Louis, MO, USA). The antibodies to human antigens obtained from Cell Signaling (Danvers, MA) included phospho-p44/p42 MAPK (Erk1/2) (T202/Y204) rabbit mAb #4377; p44/p42 MAPK (Erk1/2) Ab #9102; phospho-SAPK/JNK (Thr183/Tyr185) rabbit mAb #4668; SAPK/JNK Ab #9252; phospho-cJUN (Ser73) Ab#9164; cJUN mouse mAb #2315; phospho-p38 MAPK (Thr180/Tyr182) rabbit mAb #4511; phospho-ATF2 (Thr71) Ab #9221; ATF2 rabbit mAb #9226; phospho-AKT (Ser473) #927; AKT Ab #9272; phospho-NF-κB p65 (Ser568) rabbit mAb #3033; NF-κB p65 rabbit mAb #4764; phospho-STAT3 (Tyr705) Ab #9131; STAT3 rabbit mAb #4904; phospho-p53 (Ser15) Ab #9284; phospho-p53 (Ser20) Ab #9287; p53 Ab #9282; PARP Ab #9542; phospho-ATM (Ser1981) mouse mAb #4526; ATM rabbit mAb #2873; PD-L1 rabbit mAb #13684. The antibodies obtained from Cayman (Ann Arbor, MI) included CB1 receptor Ab #101500 and CB2 receptor Ab #101550. The secondary antibodies were conjugated to horseradish peroxidase; signals were detected using the ECL system (Thermo Scientific, Rockford, IL, USA). β-Actin served as a loading control.

### Quantitative Real-time PCR

U87MG cells were plated in 6-well plates and treated with CBD and/or radiation for the indicated time. Cells were harvested using Trizol (Thermo Fisher Scientific) after washing twice with ice-cold PBS. RNA was extracted and converted to cDNA using High Capacity cDNA reverse transcription kit (Thermo Fisher Scientific). The QPCR primer sequences are listed in Table 1.

**Table 1:** QPCR primer sequences

Target Gene	Forward Primer	Reverse Primer
IL1B	ACGCTCCGGGACTCACAGCA	TGAGGCCCAAGGCCACAGGT
IL6	TCCACAAGCGCCTTCGGTCC	GTGGCTGTCTGTGTGGGGCG
IL8	GGCCGTGGCTCTCTTGGCAG	TGTGTTGGCGCAGTGTGGTCC
TGFbeta	CTGCTGGCACCCAGCGACTC	GCAGTGGGCGCTAAGGCGAA
TNFalpha	GGCTCCAGGCGGTGCTTGTT	TGACTGCCTGGGCCAGAGGG
PDL1	CTGAACGCATTTACTGTCACGG	AGGTCTTCCTCTCCATGCAC
PDL2	CAGTGCTATCTGAACCTGTGGT	GCTGGGTCATCCAAAGGCAT
DR5	ATAAGAGCGTTCCCTACCGC	TCAGCTGAGACCAACAGCAG
FASL	ACTCCGAGAGTCTACCAGCC	CCATTCCAGAGGCATGGACC

Gene expressions were measured by Life Technologies ViiA 7 Real-Time PCR System in standard mode. QPCR was analyzed using the comparative CT method (*ΔΔCT* Method). Beta-actin was used as reference gene. For each sample, the CT value of the target gene was first normalized to the beta-actin CT value to obtain ΔCT. Then the ΔCT value was normalized to the control treatment (ctrl) within the same time-point to calculate ΔΔCT value. Showing in the graph is a 2^−ΔΔCT^ value, which indicates the fold change of target gene mRNA levels against time-point control after normalization to the reference gene.

### Suppression of cell signaling pathways by specific inhibitors

We performed inhibition of several signaling pathways using JNK1-2 inhibitor SP600125 (20 μM), MAPK p38 inhibitor SB203580 (20 μM), IKK-NF-κB inhibitor BMS345541 (20 μM); PI3K-AKT inhibitor LY294002 (40 μM); and ATM inhibitor KU60019 (1-2 μM). Western blotting with antibodies to active forms of targeted proteins was used to evaluate the effectiveness of inhibition. We also assessed changes in apoptosis levels under these conditions. All inhibitors were dissolved in DMSO as 1000X or 500X stocks; 0.1% or 0.2% DMSO was used as a control vehicle.

### Statistical analyses of data

Data from four to five independent experiments were calculated as means and standard deviations. Comparisons of results between treated and control groups were made by the Students' *t*-tests. A *p*-value of 0.05 or less between groups was considered significant.

## SUPPLEMENTARY MATERIALS FIGURES



## Abbreviations

AP1: activator protein 1
ATF2: activating transcription factor 2
ATM kinase: Ataxia Telangiectasia Mutated kinase
ATMi: ATM kinase inhibitor
CBD: cannabidiol
CHX: cycloheximide
COX2: cyclooxygenase-2
DR5: death receptor 5
ERK: extracellular signal-regulated kinase
FACS: fluorescence-activated cell sorter
FasL: Fas Ligand
GBM: glioblastoma
IκB: inhibitor of NF-κB
IKK: inhibitor nuclear factor kappa B kinase
JNK: Jun N-terminal kinase
mAb: monoclonal Ab
Luc: luciferase
MAPK: mitogen-activated protein kinase
MEK: MAPK kinase
MFI: median fluorescence intensity
NF-κB: nuclear factor kappa B
PARP: poly(ADP-ribose) polymerase
PD1: programmed cell death protein 1
PD-L1: programmed cell death protein ligand 1
PDGF: platelet-derived growth factor
PI: propidium iodide
PI3K: phosphatidylinositol 3-kinase
PTEN: Phosphatase and tensin homolog
ROS: reactive oxygen species
STAT: signal transducer and activator of transcription
TNFα: tumor necrosis factor alpha
TNFR: tumor necrosis factor receptor
TRAIL: TNF-related apoptosis-inducing ligand
TRAIL-R: TRAIL-receptor
