# Correction: Inhibition of ATM kinase upregulates levels of cell death induced by cannabidiol and γ-irradiation in human glioblastoma cells

**DOI:** 10.18632/oncotarget.27352

**Published:** 2019-12-10

**Authors:** Vladimir N. Ivanov, Jinhua Wu, Tony J.C. Wang, Tom K. Hei

**Affiliations:** ^1^ Center for Radiological Research, Department of Radiation Oncology, Vagelos College of Physicians and Surgeons, Columbia University, New York, NY 10032, USA

**This article has been corrected:** Due to errors during figure assembly, the image used in Figure 3C is incorrect. In addition, the image for Figure 3A contains accidental duplication of FACS panels. The proper Figure 3 is shown below. The authors declare that these corrections do not change the results or conclusions of this paper.

Original article: Oncotarget. 2019; 10:825–846. 825-846. https://doi.org/10.18632/oncotarget.26582

**Figure 3:**
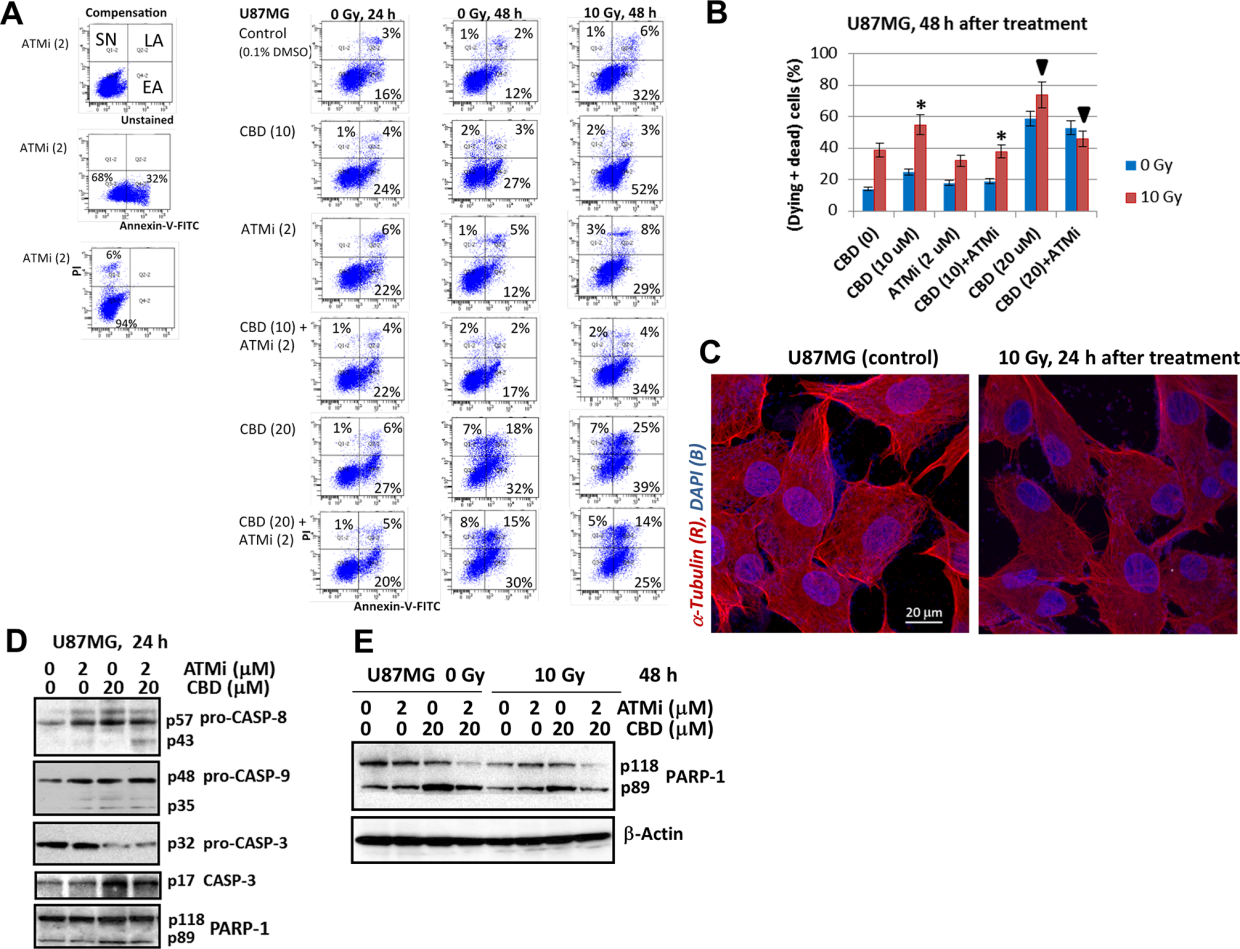
The apoptotic commitment of U87MG after treatment with CBD (10-20 μM), ATMi (2 μM) and γ-irradiation (10 Gy), alone or in combinations.

(**A** and **B**) Annexin-V-FITC and PI staining for determination of early apoptotic (EA), late apoptotic (LA) and secondary-necrotic (SN) GBM cells after indicated treatment was followed by the flow cytometry. Typical experiment (**A**) and pooled results of four independent experiments (**B**) using U87MG cells 24-48 h after indicated treatments are shown. Percentage of (dying + dead cells) included early apoptotic (EA), late apoptotic (LA) and secondary necrotic cells (SN). Error bars represent means ± S.D. (*p* < 0.05, Student’s t-test). The stars and the arrows indicate significant differences between indicated cells after specified treatment. (**C**) The images of control and irradiated U87MG after immunostaining with α-Tubulin and DAPI followed by confocal microscopy are shown. (**D** and **E**) Western blot analysis of apoptotic marker proteins 24 h and 48 h after indicated treatments of U87MG cells.

