# Supplementary material for: Inhibition of ATM kinase upregulates levels of cell death induced by cannabidiol and γ-irradiation in human glioblastoma cells
Source: Oncotarget. 2019 Jan 25;10(8):825–46. doi: 10.18632/oncotarget.26582 (PMC6368233; doi:10.18632/oncotarget.26582)
Supplement: Supplementary file 1 [file oncotarget-10-825-s001.pdf]

Inhibition of ATM kinase upregulates levels of cell death induced by cannabidiol and γ-irradiation in human glioblastoma cells

SUPPLEMENTARY MATERIALS

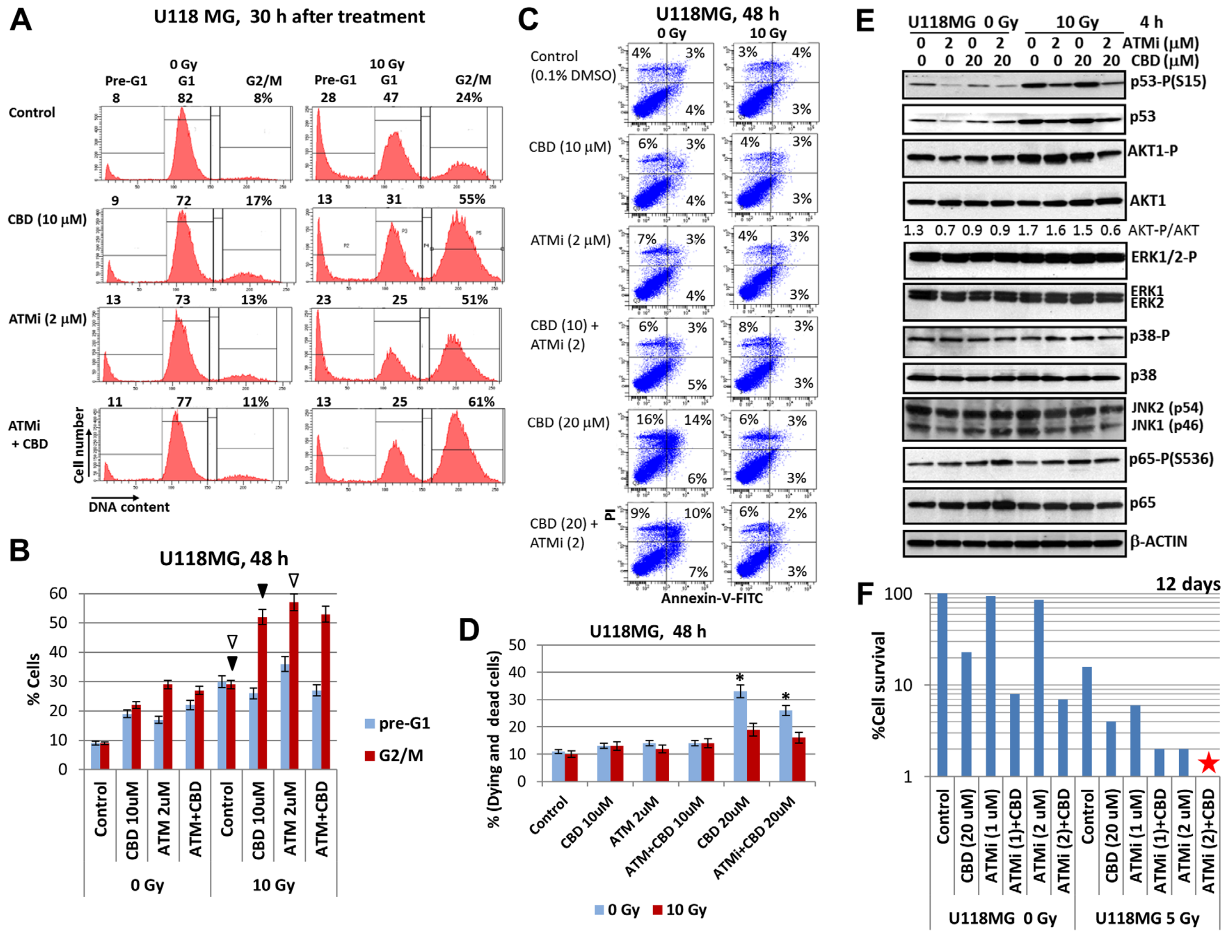

Supplementary Figure 1: Induction of cell death in U118MG glioblastoma cells following treatments with CBD, ATM inhibitor (ATMi), and γ-irradiation, alone or in combination. **(A and B)** Cell cycle-apoptosis analysis of U118MG cells 30 h after indicated treatments with CBD, ATMi and γ-radiation (10 Gy). CBD and ATMi (diluted in DMSO) were added 30 min before irradiation. Cells were stained with PI and DNA content was determined using the flow cytometry. A typical result is shown on panel **(A)**. Pooled results of four independent experiments of U118 cells 48 h after treatments are shown in panel **(B)**. Error bars represent means +S.D. ($p < 0.05$, Student's t-test). Arrows indicated significant differences in % cells at G2/M phase after specified treatments. **(C and D)** Annexin-V-FITC and PI staining was followed by the flow cytometry. Typical experiment and pooled results of four independent experiments using U118MG cells 24-48 h after indicated treatments are shown. Error bars represent means ± S.D. ($p < 0.05$, Student's t-test). **(E)** Western blot analysis of indicated signaling proteins from U118MG cells was performed 4 h after treatments with CBD (10 µM), ATMi KU60019 (2 µM), and γ-irradiation (10 Gy), alone or in combination. **(F)** Clonogenic survival assay was performed for U118MG cell cultures after indicated treatments with 0.1% DMSO (control), ATMi (1-2 µM), CBD (20 µM), γ-irradiation at 5 Gy, alone or in combination. A ratio of a number of clones of treated cells to a number of clones of control cells reflects cell survival (%). The star indicates the absence of surviving clones.

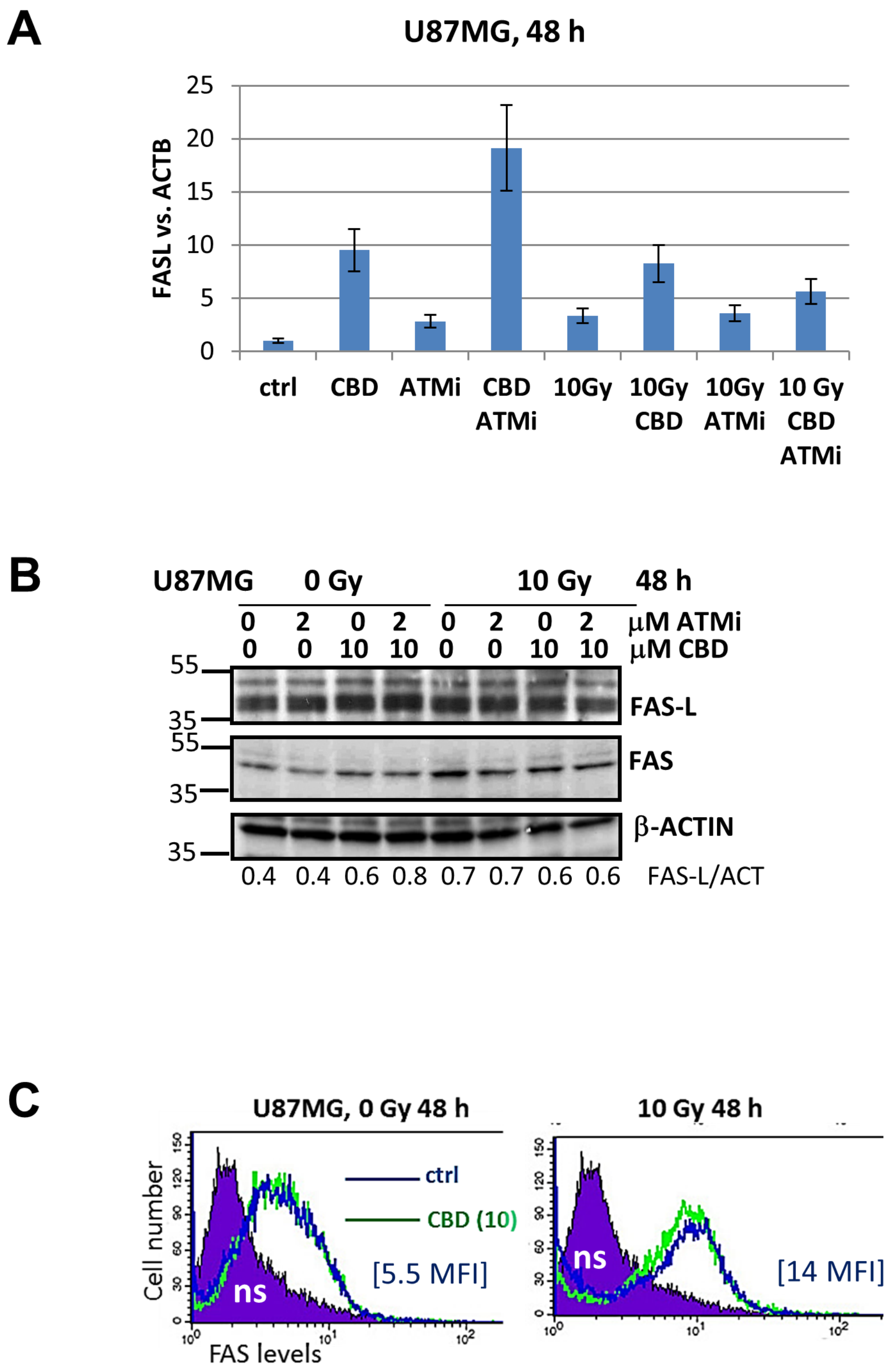

Supplementary Figure 2: FAS-L and FAS expression in U87MG GBM cells. **(A)** FASL mRNA expression was determined by quantitative real-time PCR. mRNA was isolated 48 h after treatment with 0.1% DMSO (control vehicle), CBD (10 μM), ATMi (2 μM) and γ-irradiation (10 Gy) alone or in combination. The graphs indicate the fold change of target gene mRNA levels against time-point control after normalized to the reference gene (*beta-Actin*). The pooled results of four independent experiments are presented. Error bars represent means ± S.D. ($p < 0.05$, Student's t-test). **(B)** Protein levels of FAS Ligand, FAS and β-ACTIN were determined by Western blot analysis after indicated treatments. **(C)** Surface expression of FAS after indicated treatments [control, CBD (10 μM); 10Gy, CBD+10Gy] was determined using immunostaining and the flow cytometry. Median fluorescent intensity is shown in square brackets.
